# Molecular Pathways and Circulating Biomarkers in Cerebral Cavernous Malformations—A Systematic Review

**DOI:** 10.3390/ijms27052277

**Published:** 2026-02-28

**Authors:** Hanah Hadice Karadachi, Enrique González-Gallardo, Laurèl Rauschenbach, Thiemo Dinger, Denise Zwanziger, Börge Schmidt, Anna Michel, Adrian Engel, Lisa Schock, Yuan Zhu, Oliver Gembruch, Marvin Darkwah Oppong, Ramazan Jabbarli, Yahya Ahmadipour, Ulrich Sure, Philipp Dammann

**Affiliations:** 1 Department of Neurosurgery and Spine Surgery, University Hospital Essen, Hufelandstraße 55, 45147 Essen, Germany; 2 Center for Translational Neuro & Behavioral Sciences (C-TNBS), University Duisburg-Essen, 45147 Essen, Germany; 3 Center for Familial Cavernous Malformations, Center for Rare Diseases (ESZE), University Hospital Essen, 45147 Essen, Germany; 4 Department of Neurosurgery, Mayo Clinic in Jacksonville, Jacksonville, FL 32224, USA; 5 Department of Endocrinology, Diabetology and Metabolism, Clinical Chemistry-Division of Laboratory Research, University Duisburg-Essen, University Hospital Essen, 45141 Essen, Germany; 6 Institute for Medical Informatics, Biometry and Epidemiology, University Hospital of Essen, 45147 Essen, Germany

**Keywords:** cavernoma, cerebral cavernous malformations, biomarker, plasma biomarker, molecular pathways, circulating biomarkers, endothelial dysfunction, MEKK3 signaling, systematic review

## Abstract

Cerebral Cavernous Malformations (CCMs) are low-flow vascular lesions located within the central nervous system, with a reported prevalence in the general population of 0.16–0.5%. Patients with CCMs may remain asymptomatic or present new onset symptoms such as seizures or focal neurological deficits often related to the occurrence of intracerebral hemorrhage. CCM may appear sporadic or as part of familial forms linked to mutations in the CCM-gene cluster, affecting endothelial cell integrity and triggering molecular cascades, including the MEKK3/KLF2/4 signaling pathway. Recent studies have highlighted the roles of inflammatory, angiogenic, and coagulation pathways alongside the emerging evidence of a gut–brain axis influencing microbiome-driven TLR4 signaling. This systematic review aims to describe molecular biomarkers associated with CCM pathophysiology, emphasizing their potential use as diagnostic and prognostic tools. Circulating plasma biomarkers such as CRP, vitamin D, and interleukins may reflect ongoing inflammatory and endothelial processes, while some imaging biomarkers like Quantitative Susceptibility Mapping (QSM) have shown a correlation with iron deposition and vascular leakage. Leveraging both circulating and imaging biomarkers may improve the therapeutic decision-making process. Further studies are encouraged to validate these findings and to facilitate the development of personalized, evidence-based strategies for the management of CCM.

## 1. Introduction

Cavernous Malformations (CMs; *OMIM 116860*, *603284*, *603285*) are the second most common cerebral vascular malformations, characterized by low-flow, hemorrhagic lesions in the brain and spinal cord, affecting 0.16–0.5% of people. They are composed of tightly clustered, abnormally dilated, leaky capillary channels lined by a single endothelial layer with impaired tight junctions. Unlike normal vessels, CMs lack structural components like pericytes and astrocyte foot processes and are surrounded by a thick basal membrane without intervening brain tissue, making them angiographically occult [1,2,3].

They may be single or multiple and show variable clinical behavior, from asymptomatic to seizures, neurological deficits, or hemorrhage. Symptoms and severity are unpredictable [2,3]. Spinal cavernous malformations (SCMs), histologically identical but rare, account for about 5% of all CMs and 5–12% of intraspinal vascular malformations, mainly affecting the thoracic and cervical spine. Familial SCMs occur in up to 12% of cases and often coexist with intracranial lesions [4,5,6].

CCMs can be classified into sporadic (sCCM) and familial (fCCM) forms. Sporadic CCMs account for approximately 80% of cases and typically present as solitary lesions, whereas fCCM follows an autosomal dominant inheritance pattern, is less prevalent, and is characterized by multifocal lesions that increase in number and size over time [1,7].

Familial CCM is most commonly associated with loss-of-function mutations in the *KRIT1 (CCM1; 116860)*, *MGC4607 (CCM2; 603284)*, and *PDCD10 (CCM3; 603285)* genes, accounting for approximately 80% of cases, while the remaining cases are attributed to yet unidentified genetic or molecular mechanisms [3,8,9].

Despite advances in genetic characterization, a full understanding of the molecular mechanisms behind CCM initiation, progression, and severity remains elusive. Loss of CCM-complex proteins impacts angiogenic signaling, vascular homeostasis, cytoskeletal regulation, and inflammation. Environmental and systemic factors also influence CCM-related pathways and may serve as biomarkers for disease activity and outcome.

This study explores the pleiotropic roles of CCM proteins in disease pathophysiology. We systematically reviewed literature to identify circulating molecular biomarkers that could inform personalized therapy, improve prognosis, and guide CCM management decisions. While focusing on circulating biomarkers, we included tissue, genetic, and imaging markers relevant to disease mechanisms and clinical stratification.

By linking molecular pathways to circulating and imaging biomarkers, this review adopts a molecular medicine approach to connect CCM pathophysiology to measurable biological signatures with translational potential.

### 1.1. CCM Genes Germline Mutations

Recent genetic linkage studies have identified three CCM loci on chromosomes *7q (CCM1)*, *7p (CCM2)*, and *3q (CCM3)*. A founder effect is evident in Hispano-American patients, with most familial CCM cases linked to CCM1. In Caucasian families, the distribution is more balanced: about 40% linked to *CCM1*, 20% to *CCM2*, and 40% to *CCM3*. The *CCM1* gene has 16 exons that encode *KRIT1*, which is involved in Rap1 and integrin signaling. *CCM2* has 10 exons for Malcaverin/MGC4607, a scaffolding protein in the p38 MAPK pathway. *CCM3* contains 7 exons encoding PDCD10, which is involved in apoptosis regulation. Focal *CCM (fCCM)* mainly results from heterozygous loss-of-function mutations—often nonsense, frameshift, or splice-site mutations—though these are a minority of genetic alterations (Figure S1) [1,6,9].

### 1.2. The “Two-Hit” Mechanism

Sporadic and familial CCM cases generally differ in lesion burden: sCCM usually involves solitary lesions, while *fCCM* presents with multiple lesions and an earlier onset. Some forms of *fCCM* also show high variability in clinical manifestations. According to Fauth et al., early fulminant cerebral hemorrhage has been reported in patients with *CCM3* mutations and appears more common in this subgroup compared to *CCM1* or *CCM2* carriers [10]. Notably, some patients without a known family history may still develop multiple lesions due to a de novo germline mutation, indicating a possible secondary event involving the wild-type allele in CCM pathophysiology. This idea aligns with the “Two-Hit” hypothesis, which suggests that an additional somatic mutation inactivating the remaining wild-type allele could lead to lesion formation. Supporting this, several studies have shown that ionizing radiation is a strong trigger for CCM development. Notably, patients with radiation-induced CCMs often have multiple lesions, unlike sporadic cases without prior radiation exposure, where solitary lesions are common norm [1,6,11,12].

The “Two-Hit” model in CCM pathology has been difficult to validate due to technical limitations, cellular heterogeneity of lesions, and the scarcity of endothelial cells lining abnormal capillaries. Nonetheless, evidence has emerged. Gault et al. found a somatic 34-nucleotide deletion in *CCM1* in a patient with a germline CCM1 mutation, confirmed by allele-specific PCR and sequencing, supporting the “Two-hit” mechanism since the mutation was absent in blood DNA and RNA [13,14]. Studies also show that patients with germline mutations in *CCM1*, *CCM2*, or *CCM3* lack the corresponding CCM protein in endothelial cells of lesions, indicating bi-allelic mutation-induced loss of function [14]. However, Pagenstecher et al. [15] noted the difficulty in detecting loss-of-heterozygosity (LOH) due to the limited number of affected cells and the mosaic nature of the endothelium, complicating molecular studies. Akers et al. demonstrated somatic mutations across all inherited CCM types, suggesting CCM genes act like tumor suppressors needing bi-allelic inactivation for lesion formation. Further research indicates CCM proteins are crucial for vascular endothelial integrity; their loss disrupts cell junctions, vascular stability, and responses to stress and inflammation, leading to hemorrhagic lesions [9,16].

While the importance of a second-hit mechanism in CCM development has been well established, it remains unclear whether there is a critical time window during which CCM protein inactivation may induce a CCM-specific phenotype. To address this question, Pilz et al. investigated the role of *CCM1* in non-endothelial cell types and the importance of exogenous triggers in CCM formation. They analyzed the gene expression profile in endothelial cells derived from a *CCM1*-knockout induced pluripotent stem cell (iPSC) model *(CCM1^−^/^−^ iPSC)* to determine whether *CCM1* deficiency also alters gene expression in *iPSCs* and early mesoderm progenitor cells (eMPCs). After comparing the gene expression profiles of *CCM1^+^/^+^* and *CCM1^−^/^−^ iPSC*-derived endothelial cells, they found that loss of CCM1 expression led to substantial changes in gene expression, including upregulation of KLF2, KLF4, and other CCM-associated genes. However, there were a few differences in gene expression between *iPSCs* and *eMPCs*. The results indicate that CCM1 deficiency does not necessarily result in a cellular or molecular phenotype until the cells differentiate into the endothelial lineage. Moreover, *CCM1^−^/^−^* precursor cells may exist that remain silent until entering the endothelial differentiation [17].

Furthermore, non-genetic third hits are believed to play a critical role in the genesis of CCM. It has also been proposed that the establishment of a *CCM1*-knockout-specific gene expression pattern may be enabled by exogenous factors, such as cytokines and growth factors, which induce endothelial cell differentiation. In this regard, Abdelilah-Seyfried et al. proposed that non-genetic “third hits,” such as hypoxia and angiogenic factors, may also contribute to CCM pathogenesis by activating quiescent CCM-deficient cells [18].

### 1.3. The Gain-of-Function Mutations as a Consequence of CCM Complex Loss-of-Function

The Two-hit model elucidates CCM disease through a mechanism akin to cancer, wherein the accumulation of somatic mutations results in loss-of-function (LOF) within the CCM gene complex, which functions as a suppressor of vascular malformations. Nonetheless, this model alone fails to account for the abrupt and rapid enlargement of individual CCM lesions, which often lead to stroke or seizure. Investigations utilizing murine models have yielded critical insights into this limitation. Homozygous *CCM1*-deficient mice generally perish during mid-gestation due to profound defects in vascular morphogenesis. Similarly, endothelial-specific deletion of *CCM2* hampers embryonic angiogenesis, resulting in lethality at mid-gestation. However, contrary to human CCM, these vascular lesions do not develop following gene deletion in neuronal or smooth muscle cells in adult mice. This discrepancy has fostered the hypothesis that additional proliferative signals may be requisite for lesion formation and progression in humans. Empirical evidence supports this hypothesis; recent studies have demonstrated that endothelial cells isolated from human CCM lesions frequently harbor gain-of-function (GOF) mutations, notably in the oncogene *PIK3CA*. These mutations induce proliferative signaling pathways, thereby indicating a dual mechanism whereby both LOF mutations in CCM genes and GOF mutations in oncogenic pathways collectively contribute to lesion formation, growth, and clinical deterioration. This emerging model broadens the understanding of CCM pathogenesis and suggests potential therapeutic targets within proliferative signaling pathway inhibition [19,20,21,22,23].

GOF mutations in the *PIK3CA* gene drive cell proliferation in cancer and are linked to venous and lymphatic malformations in endothelial cells. Recent studies found somatic *PIK3CA* GOF mutations in both sporadic and familial CCMs across all genotypes, indicating the role of oncogenic pathways in CCM development. A key mechanism involves MEKK3 hyperactivation, which increases KLF2 and KLF4 expression in endothelial cells, promoting pro-angiogenic and inflammatory phenotypes that support abnormal vascular growth. *PIK3CA* GOF mutations worsen this by causing hyperproliferative vessels with reduced pericyte coverage and lower arteriovenous differentiation markers, resulting in vascular instability. These changes are characteristic of *PIK3CA*-Related Overgrowth Spectrum (PROS) disorders, where vascular malformations are common [19,24,25].

Previous studies show sporadic CCMs often lie near a Developmental Venous Anomaly (DVA), a common vascular malformation with 6–14% prevalence, mostly before age 20. Albdulrauf et al. found an adjacent DVA in 24–36% of sporadic CCM cases, especially in surgical cases, suggesting a possible link. Conversely, fCCMs are not associated with DVAs, indicating DVA is not necessary for CCM development. These findings imply DVAs may predispose to sCCMs by affecting venous flow, vascular stress, or creating an environment for lesion formation [25,26,27,28].

Snellings et al. studied genetic interactions among somatic mutations in *KRIT1*, *CCM2*, *PDCD10*, *MAP3K3*, and *PIK3CA*, involved in CCM. They found that fCCM patients lacked MAP3K3 mutations, while sCCM cases could have mutations in *MAP3K3*, *KRIT1*, *CCM2*, or *PDCD10*. Each lesion usually had mutations in only one gene, indicating any mutation is enough for CCM without needing others. They proposed a two-step model: initial mutation causes clonal expansion of endothelial cells, forming a lesion. As the clone grows, the chance of a second mutation increases with more mutated cells. Similar process occurs in DVAs, where *PIK3CA* GOF mutations may trigger expansion. *PIK3CA* mutations in DVAs suggest they could be precursors to sporadic CCMs, explaining the link. DVAs are rarely associated with fCCM, likely due to inherited germline mutations in familial cases. Since fCCM patients already have a CCM gene mutation, the chance of a second somatic mutation in another CCM gene is higher than a *PIK3CA* GOF mutation. More LOF mutations are possible in CCM genes than GOF mutations in *PIK3CA*, explaining different pathways in sporadic and familial CCM development [28].

Studies in zebrafish have demonstrated a high prevalence of somatic activating mutations in both *MAP3K3* and *PIK3CA* in endothelial cells of sCCMs, revealing distinct genetic signatures across different subgroups. *MAP3K3* encodes MEKK3 (mitogen-activated protein kinase kinase 3), a critical regulator of several signaling pathways implicated in CCM pathogenesis. Hong et al. identified activating mutations in both *MAP3K3* and *PIK3CA* in 90.1% of sporadic CCM cases, suggesting that activation of the MAPK signaling pathway plays a key role in both sporadic and familial CCM development. GOF mutations in MAP3K3 enhance MAPKK3 kinase activity, leading to the hyperactivation of several downstream pathways, including ERK1, ERK2, ERK5, JNK, and p38 MAPK. In brain endothelial cells, activation of the p58 pathway induces increased expression of apoptotic factors, as observed both in vitro and in murine lesions. This suggests that p38-mediated signaling contributes to the abnormal development of blood vessels in CCM lesions.

Experimental work in mice suggests that endothelial MP3K3 activation can promote CCM-like vascular lesion formation early in life; lesion burden may subsequently decline as p38-dependent endothelial apoptosis becomes prominent, implying that sustained lesion growth likely requires concurrent pro-survival/proliferative signaling. In this context, PI3K pathway activation can support endothelial proliferation and attenuate p38-mediated apoptosis, consistent with a model in which proliferative and anti-apoptotic cues cooperate to maintain lesion progression. Complementing these mechanistic observations, human genetic studies have identified somatic *MAP3K3* mutations that define a subset of sporadic CCM: Weng et al. reported a *MAP3K3*-mutant subclass of CCM, and Ren et al. showed that endothelial-restricted expression of the *MAP3K3 p.Ile441Met* variant is sufficient to induce both cerebral and spinal cord cavernous malformations in vivo. Collectively, these data support a convergent role of *MAPK* (MAP3K3-p38) and PI3K signaling in CCM biology, and hence, motivate studies on biomarkers targeting molecular pathways reflecting endothelial activation, survival, and lesion activity [21,24,25,29,30,31,32,33,34].

In mice, CCM lesions arise after endothelial loss of CCM genes during early postnatal development, whereas lesion development or persistence in adult models may require additional permissive signaling, inducing activation of PI3K. In humans, CCMs can arise even in the absence of *PIK3CA* mutations, particularly during periods of rapid growth or in response to various environmental factors. These findings suggest that *PIK3CA* mutations may be particularly critical for sustaining CCM lesions driven by *MAP3K3* activation, as they help counteract apoptosis induced by MAPK pathway hyperactivation. This implies that *MAP3K3*-mutant CCMs may represent a distinct molecular subclass of the disease. Consequently, targeting PI3K signaling could serve as a promising pharmacological approach for treating patients with *MAP3K3*-driven CCMs [20,25,29].

### 1.4. Molecular Signaling Pathways Involved in CCM Lesion Formation and Progression

**Figure 1:**
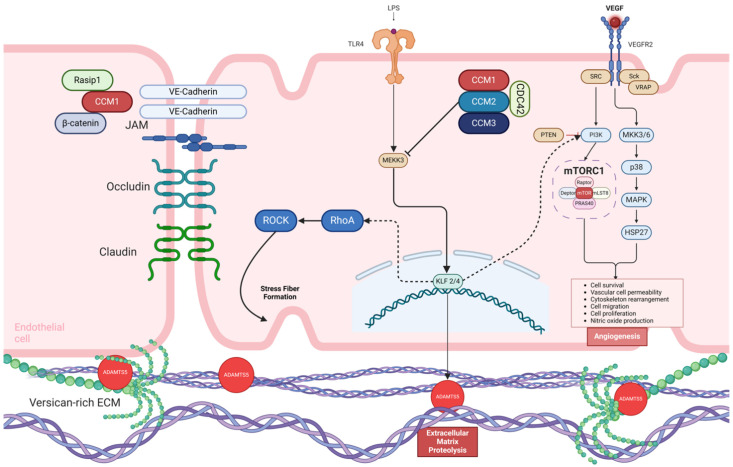
Molecular signaling pathways involved in Cerebral Cavernous Malformations. The CCM complex is composed of three different proteins, CCM1/KRIT1, CCM2, and CCM3 (PDCD10), which in normal conditions acts as an inhibitor of MAP3K3 Kinase, MEKK3. Proinflammatory signaling through TLR-4 serves as an activating input to MEKK3 signaling. In CCM disease, the loss of function of any of the components in CCM complex causes a subsequent release of MEKK3 in response to TLR-4 activation, leading to an upregulation of different transcription factors mediated at the nucleus by KLF2/4. The downstream KLF2/4 activation triggers cell autonomous pathways through PI3K signaling and RhoA/ROCK pathway, as well as cell non-autonomous effects through activation of ADAMTS5, which causes a subsequent cleavage of versican polymers and other extracellular matrix components. Activation of RhoA/ROCK signaling pathway results in stress fiber formation causing a dysregulation of endothelial cell junctions, thus increasing vascular permeability. VEGFR2 activation is another proposed mechanism for PI3K-mTOR signaling upregulation, resulting in the synthesis of various angiogenic factors. **CCM1/KRIT1**, Krev Interaction Trapped Protein 1; **CCM2/MGC4607**, Malcaverin Protein; **CCM3/PDCD10**, Programmed Cell Death 10 Protein; **CDC42**, Cell Division Control Protein 42; **MEKK3**, dual specificity mitogen-activated protein kinase kinase 3; **TLR-4**, Toll-Like Receptor-4; **LPS**, Lipopolysaccharide; **VEGF**, Vascular Endothelial Growth Factor; **VEGFR2**, Vascular Endothelial Growth Factor Receptor-2; **SRC**, Proto-oncogene Tyrosine-protein Kinase; **Sck**, Shc-related Adaptor Protein; **VRAP**, VEGF Receptor-Associated Protein; **PI3K**, Phosphoinositide 3-Kinase; **PTEN**, Phosphatase and Tensin Homolog; **p38**, Mitogen-Activated Protein Kinase-38; **MAPK**, Mitogen-Activated Protein Kinase; **HSP27**, Heat Shock Protein-27; **mTORC1**, Mammalian Target of Rapamycin Complex-1; **mTOR**, Mammalian Target of Rapamycin; **Raptor**, Regulatory-Associated Protein of mTOR; **Deptor**, Domain-Containing mTOR-interacting Protein; **mLST8**, Target of Rapamycin Complex Subunit LST8/Mammalian Lethal with SEC13 Protein-8; **PRAS40**, Proline-Rich Akt Substrate of 40kDa; **KLF-2/4**, Krüppel-Like Factor-2/4; **RhoA**, Ras Homolog Family Member A; **ROCK**, Rho-Associated Protein Kinase; **Rasip-1**, Ras-Interacting Protein-1; **ADAMTS5**, A Disintegrin and Metalloproteinase with Thrombospondin Motifs-5, **ECM**, Extracellular Matrix; **JAM**, Junctional Adhesion Molecule. Adapted from Snellings D et al. 2022 [28].

Understanding the complex cellular and molecular mechanisms underlying CCM formation requires examining the heterotrimeric adaptor complex formed by *KRIT1*, *CCM2*, and *PDCD10*, which regulates endothelial cell function. It is now well established that the loss of any of these non-homologous proteins in endothelial cells of the developing heart in zebrafish and mice leads to upregulation of KLF2, KLF4, ADAMTS4, and ADAMTS5 expression due to hyperactivation of the MEKK3 signaling pathway. The CCM2 protein directly interacts with MEKK3, a kinase that regulates the expression of KLF2 and KLF4 in cultured endothelial cells. Zhou et al. demonstrated that *MAP3K3* haploinsufficiency could prevent early embryonic lethality caused by pan-endothelial loss of KRIT1, suggesting that excessive MEKK3 signaling induces abnormal KLF2, KLF4, and ADAMTS4 expression, contributing to lesion formation. Their study further identified KLF2 and KLF4 as critical downstream targets of MEKK3 activation in mouse models, establishing this pathway as a central driver of CCM pathogenesis. They also analyzed resected lesions from sporadic and familial CCM patients and found that disrupting the interaction between CCM2 and MEKK3 was sufficient to induce fCCM, emphasizing the pivotal role of KLF2/4 upregulation in the disease’s molecular pathogenesis (Figure 1) [35,36].

Beyond this point, two effector pathways have been implicated in the pathophysiology of CCM: Rho signaling and ADAMTS proteolytic activity [35].

### 1.5. The Role of Rho/ROCK and ADAMTS in CCM Vascular Lesions

The RhoA/ROCK signaling pathway plays a crucial role in regulating various cellular processes, including cell contractility, migration, proliferation, and differentiation and the integrity of cell–cell junctions. ROCK, a serine/threonine kinase, functions as a downstream effector of the GTPase RhoA. Like many other protein-kinases, ROCK has two main isoforms, ROCK 1 and ROCK 2, with tissue-specific expression patterns. ROCK2 is the predominant isoform in brain endothelial cells, where it directly influences the integrity of inter-endothelial cellular junctions, playing a critical role in maintaining vascular barrier function [37].

Under normal conditions, the CCM protein complex acts as a negative regulator of RhoA/ROCK signaling. However, LOF mutations of any of the CCM-complex components result in disinhibition of RhoA-dependent ROCK activity, triggering downstream cytoskeletal changes. This disinhibition promotes the formation of stress fibers: contractile structures composed of actin and myosin filaments anchored to the extracellular matrix (ECM) through focal adhesions. The formation of stress fibers has several pathological consequences. Their contractile nature disrupts cell–cell junctions in the cerebrovascular endothelium, increasing vascular permeability and heightening the risk of intracerebral hemorrhage. In addition, the altered cytoskeletal dynamics driven by ROCK activation can promote cell migration and angiogenesis in the presence of angiogenic factors such as Vascular Endothelial Growth Factor (VEGF). This abnormal angiogenesis is thought to contribute to the development and progression of CCM lesions by facilitating the expansion of malformed vascular structures [20,35,36,37].

In vitro studies have shown that endothelial cells with *CCM1* or *CCM2* silencing tend to adopt a senescence-associated secretory phenotype (SASP), characterized by increased production of proinflammatory cytokines, chemokines, and ECM metalloproteinases. This phenotypic shift is closely linked to *ROCK* activation, underscoring its role in driving inflammation and tissue remodeling in CCM pathogenesis. The two *ROCK* isoforms play distinct but complementary roles in promoting the inflammatory microenvironment associated with CCM lesions. *ROCK2* is the primary isoform responsible for inducing leukocyte chemotaxis, facilitating immune cell recruitment to the affected vascular endothelium. In parallel, *ROCK1* enhances the activity of matrix metalloproteinases (MMPs), leading to ECM degradation and further tissue remodeling. This dual action contributes to a proinflammatory milieu, amplifying local inflammation and vascular instability, which are hallmarks of CCM lesion progression [37,38].

The Disintegrin-like and Metalloproteinase with Thrombospondin Motif (ADAMTS) family includes 19 proteases that regulate the structure and function of ECM proteins. Among them, ADAMTS5, a key protease that cleaves the proteoglycan versican, plays a significant role in ECM remodeling. Its expression is regulated by MEKK3 signaling, and its dysregulation has been implicated in CCM pathogenesis. Hong et al. demonstrated that endothelial-specific loss of ADAMTS5 in mouse models leads to increased versican accumulation in white matter, a region where CCMs commonly form, significantly reducing CCM formation. This suggests that ADAMTS5-mediated versican cleavage contributes to the structural changes necessary for lesion development. Conversely, GOF mutations in ADAMTS5 were shown to induce vascular changes characteristic of early CCM lesions, even in the absence of increased MEKK3-KLF2/4 signaling. Additionally, ADAMTS5 overactivity strongly synergized with KRIT1 deficiency, exacerbating CCM formation. These findings highlight ADAMTS5 as a critical mediator of vascular remodeling and a potential therapeutic target in CCM disease [39,40].

In summary, the production of stress fibers and proinflammatory cytokines mediated by *RhoA/ROCK* disinhibition, coupled with increased proteolytic activity in the ECM, leads to the destabilization of adherent cellular junctions. This disruption increases vascular permeability, contributing to the heightened risk of bleeding, a hallmark of CCM disease [37,38,41,42,43].

### 1.6. The Gut–Brain Axis in CCM Disease

It is well established that fCCM arises from germline and heterozygous LOF mutations in any of the three CCM genes, activating the aforementioned molecular pathways involved in CCM disease. Denier et al. conducted a comparative study examining the clinical and molecular characteristics of individuals with *CCM1, CCM2,* and *CCM3* mutations. They observed that carriers of *PDCD10 (CCM3)* mutations were more likely to develop symptoms before the age of 15 and experienced ICH at a younger age compared to those with *KRIT1/CCM2* mutations. Further supporting these findings, Shenkar et al. reported that *PDCD10* mutation carriers exhibited more severe clinical manifestations and a significantly greater lesion burden in both human and murine models than individuals with other CCM mutations. These observations suggest that *PDCD10* mutations may drive CCM development through distinct, possibly independent, mechanisms, indicating a unique pathological role for this gene in disease progression [44,45,46].

Emerging evidence over the past decade suggests that brain-resident and peripheral immune cells play a crucial role in mediating communication between the gut microbiota and brain tissue. According to Fung and colleagues, the gut microbiota can influence and regulate the developmental functions of both microglia and astrocytes, which are essential for neurophysiological processes such as neural development, neurotransmission, Central Nervous System (CNS) immune activation, and the maintenance of blood–brain barrier integrity. This bidirectional interaction, commonly referred to as the “Gut–Brain Axis,” has been implicated in various neurological disorders, including stroke, Alzheimer’s disease, and Parkinson’s disease. Several mechanisms have been proposed to explain this connection. These include microbiota-derived metabolites that affect brain function, modulation of local immune cells in the gut that may migrate to CNS vasculature, and direct communication mediated by circulating neuro-humoral factors. Despite these advances, the precise molecular and cellular mechanisms underlying the gut–brain axis remain poorly understood for many of these neurological disorders. Further research is needed to clarify how specific gut microbiota components influence brain health and disease progression through immune and neurovascular pathways [45,47,48].

Tang et al. demonstrated that lipopolysaccharide (LPS), a key component of the outer membrane of Gram-negative bacteria (GNB) in the gut microbiome, accelerates CCM disease progression by activating the Toll-like receptor 4 (TLR4) on brain endothelial cells. This activation upregulates MEKK3 signaling, promoting endothelial dysfunction and CCM lesion formation, supporting gut–brain-mediated mechanisms in CCM development. In subsequent research, the same group found that *PDCD10*-dependent changes in the colonic gut barrier contribute to more severe clinical phenotypes observed in individuals with *PDCD10* germline heterozygosity compared to those with *KRIT1* or *CCM2* mutations. Using a neonatal mouse CCM model, they further demonstrated that the gut microbiome composition plays a paramount role in lesion formation, with animal subjects harboring greater numbers of GNB in the colon exhibiting greater CCM lesion formation [45,49].

**Figure 2:**
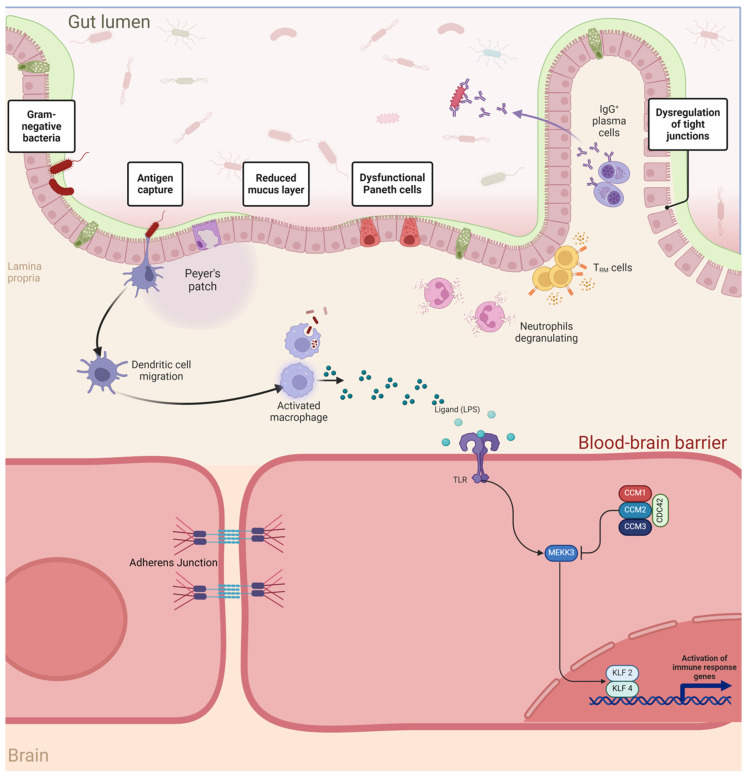
Proposed Gut–Brain Axis Mechanism of Cerebral Cavernous Malformation Disease. The loss of function of PDCD10 in intestinal endothelial cells is associated with swollen goblet cells, resulting in a marked reduction of the mucus barrier. This suggests that a primary mechanism by which PDCD10 deficiency in intestinal endothelial cells might accelerate CCM formation is through loss of the mucus layer that normally prevents Gram-negative bacteria, and GNB-LPS, from reaching the gut endothelial wall and translocating to the blood stream, resulting in elevated TLR4 agonist activity, which in turn promotes MEKK3 signaling in brain endothelial cells. ***CCM1/KRIT1***, Krev Interaction Trapped Protein 1; ***CCM2/MGC4607***, Malcaverin Protein; **CCM3/PDCD10**, Programmed Cell Death 10 Protein; **CDC42**, Cell Division Control Protein 42; **MEKK3**, dual specificity mitogen-activated protein kinase kinase 3; **TLR-4**, Toll-Like Receptor-4; **LPS**, Lipopolysaccharide; **KLF-2/4**, Krüppel-Like Factor-2/4. Adapted from Snellings D et al. 2022 [28].

The aforementioned studies suggest that the more aggressive clinical course observed in *fCCM* among *PDCD10* mutation carriers is not solely due to a unique signaling role in brain endothelial cells or a specific effect on the gut microbiome. Instead, it may reflect a previously unrecognized role for *PDCD10* in the integrity of the gut barrier. A critical component of the colonic gut barrier is the mucus layer, produced by goblet cells, which physically separates gut bacteria from the intestinal epithelium. Loss of function (LOF) mutations in *PDCD10* in intestinal endothelial cells have been linked to swollen goblet cells and a significant reduction in the mucus barrier. This disruption compromises the gut barrier’s protective function, allowing Gram-negative bacteria (GNB) and their endotoxin lipopolysaccharide (LPS) to reach the gut endothelial wall and potentially translocate into the bloodstream. The resulting systemic circulation of LPS acts as a Toll-like receptor 4 (TLR4) agonist, triggering MEKK3 signaling in brain endothelial cells. This pathway activation promotes cerebrovascular inflammation and CCM lesion formation, providing a mechanistic explanation for the more severe phenotype observed in *PDCD10* mutation carriers (Figure 2) [45,49]. Polster et al. demonstrated that individuals with CCM disease exhibit a distinct microbiome composition compared to healthy individuals. Their study further revealed that CCM patients with varying clinical disease characteristics possess different bacterial microbiota profiles, suggesting a potential link between microbiome composition and disease severity or progression. Additionally, they proposed that combining plasma biomarker analysis with stool microbiome profiling could have diagnostic value. This integrated approach may help identify specific microbial signatures and systemic markers associated with CCM, offering the potential for more precise disease monitoring and personalized therapeutic strategies [48].

### 1.7. CCM Disease Progression and Hemorrhage

Clatterbuck et al. described the dynamic behavior of CCMs, noting their potential for de novo formation, enlargement, and even regression. According to Ozgen et al., CCMs can range in size from just a few millimeters to several centimeters, with an average of 1.4 cm in diameter, though some lesions may exceed these dimensions. This dynamic nature underlies neurological manifestations commonly associated with the disease. Clinically, CCMs often present with epileptic seizures or, in the event of hemorrhage, focal neurological deficits. While some studies suggest that fCCM cases have higher symptomatic hemorrhage rates than sporadic cases, the findings remain inconsistent. The reported annual risk of symptomatic hemorrhage varies widely among studies, ranging from 0.25% to 22.9% per patient-year, reflecting differences in study populations and methodologies. Despite this variability, it is generally accepted that all CCMs experience occult bleeding, as evidenced by the hallmark appearance of a hemosiderin halo surrounding the lesion on MRI. This finding supports the notion that subclinical hemorrhages are a defining feature of CCM pathology, contributing to its progressive nature and associated clinical risks [1,50,51,52].

Immunohistochemical analyses have shown that the primary anatomical basis for bleeding in CCM lesions is the loss of cell–cell junction integrity combined with increased cytoskeleton contractility in low-flow areas. This structural instability results directly from the LOF of the CCM-protein complex. As previously discussed, LOF mutations in *KRIT1*, *CCM2*, or *PDCD10* activate a pathological signaling cascade that upregulates the transcription factors KLF2 and KLF4. This process is linked to increased expression of both angiogenic and anticoagulant factors, contributing to lesion growth and risk of hemorrhage. Among these factors, Vascular Endothelial Growth Factor (VEGF) plays a central role. The combined loss of *KRIT1* and *PDCD10* results in heightened VEGF expression, which activates VEGFR2 on endothelial cells. VEGFR2 activation increases vascular permeability by promoting endothelial monolayer leakage, stress fiber formation, and cellular migration. It also induces the phosphorylation of VE-cadherin and β-catenin, disrupting adherens junctions, and facilitates angiogenesis and lesion progression. DiStefano et al. demonstrated that VEGFR2 inhibition effectively blocked microvascular permeability and significantly reduced hemorrhage occurrence in murine CCM models. These findings underscore the therapeutic potential of targeting VEGFR2 signaling as a strategy for stabilizing CCM lesions and mitigating their clinical impact. This mechanism provides a promising avenue for developing future treatments aimed at reducing bleeding risks and slowing CCM disease progression [53,54].

**Figure 3:**
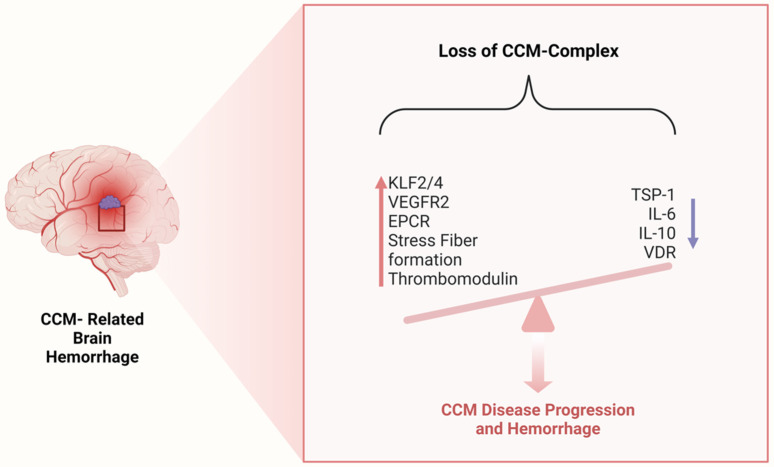
Underlying mechanisms associated with CCM-Related Hemorrhage. **KLF-2/4**, Krüppel-Like Factor-2/4; **VEGFR2**, Vascular Endothelial Growth Factor Receptor-2; **EPCR**, Endothelial Protein C Receptor; **TSP-1**, Thrombospondin-1; **IL-6**, Interleukin-6; **IL-10**, Interleukin-10; **VDR**, Vitamin D Receptor.

López-Ramírez et al. discovered that the upregulation of KLF2 and KLF4 in endothelial cells leads to the suppression of *Thbs1*, the gene encoding thrombospondin 1 (TSP1), a potent endogenous inhibitor of angiogenesis. Under normal conditions, TSP1 limits excessive VEGFR2 signaling triggered by the loss of endothelial *KRIT1*, stabilizing tight junctions and preventing capillary dilation. Its suppression in CCM lesions exacerbates vascular instability, contributing to lesion growth and increased permeability. In subsequent studies, the same group reported that CCM-related hemorrhage is closely linked to the upregulation of thrombomodulin (TM) and the endothelial protein C receptor (EPCR), key regulators of the anticoagulant pathway. Both receptors were found to have elevated soluble plasma levels, correlating with an increased risk of hemorrhage in *PDCD10*-deficient mice. These findings led to the conclusion that the CCM microenvironment represents a pathological combination of anticoagulant vascular properties and heightened angiogenic activity, driven by dysregulated endothelial signaling. This imbalance creates a vascular domain prone to leakage, ultimately contributing to recurrent hemorrhages in CCM lesions. Understanding these mechanisms highlights potential therapeutic targets aimed at restoring vascular homeostasis by modulating both proangiogenic and anticoagulant pathways [52,53] (Figure 3).

In contrast to the theory that increased angiogenic activity is the primary driver of CCM-related ICH, growing evidence suggests that genes and proteins related to the coagulation cascade may also play a significant role in influencing the brain vasculature, contributing to neurological complications such as hypoxia within CCMs. Globisch et al. demonstrated that inflammation within CCMs recruits neutrophils that form neutrophil extracellular traps (NETs), which, together with pro-coagulant factors, promote immunothrombosis. This process involves thrombus formation as part of an inflammatory response, potentially exacerbating vascular instability. Their study also identified distinct vascular regions within CCM lesions prone to different pathological outcomes: “hot” regions, characterized by a high propensity for thrombus formation, and “cold” regions, more susceptible to bleeding. This vascular heterogeneity indicates that CCM lesions are dynamic environments where coagulation imbalances contribute to both hemorrhage and cerebral hypoxia, complicating the clinical presentation of the disease [55].

Under normal physiological conditions, endothelial cells in the brain vasculature create a smooth nonadherent surface that prevents platelet adhesion and inhibits activation of the coagulation cascade. However, disturbances in vascular homeostasis can induce a switch from an anticoagulant to a prothrombotic state in these cells. In response to injury, endothelial cells expose and release von Willebrand factor (vWF), which is typically stored in Weibel–Palade bodies. Upon release, vWF uncoils and binds to exposed subendothelial components, mediating platelet adhesion and activation. This creates a positive feedback loop that stimulates further vWF release from activated platelets. Accumulation of vWF has been observed within the lumen of CCMs in human subjects, and in vitro models of CCM show increased vWF production in endothelial cells lacking CCM1 or CCM3. These cells form long vWF-strings, suggesting that vWF release is a downstream consequence of LOF in CCM genes. In this context, Globisch et al. proposed that endothelial cells deficient in CCM function might actively recruit and activate platelets, contributing to the early stages of CCM development [55,56,57,58].

Endothelial dysfunction in CCM lesions often leads to blood stasis due to altered vessel architecture. CCM lesions typically exhibit an irregular, tortuous vascular phenotype, similar to the pathological dilations found in cerebral aneurysms. This abnormal structure results in disorganized and turbulent blood flow, reducing wall shear stress and promoting the activation of prothrombotic factors. The formation of blood clots within these malformed vessels can obstruct oxygen delivery to adjacent parenchymal cells. In response, these cells may upregulate pro-angiogenic and tissue remodeling factors in an attempt to restore tissue homeostasis. However, this compensatory mechanism can worsen lesion growth by stimulating aberrant angiogenesis and further vascular instability. Moreover, the vascular dysfunction characteristic of CCM lesions may impair the activation of the fibrinolytic system, leaving blood clots unresolved and contributing to long-term partial vessel occlusions. This persistent obstruction could result in localized ischemia and chronic hypoxia within CCM lesions, exacerbating various pathological processes, including inflammation, vessel remodeling, and hemorrhage [1,57,59,60].

The development of prothrombotic microenvironment in CCM lesions results from interactions between endothelial cells and non-cell autonomous effectors. Senden et al. demonstrated that endothelial cells, when exposed to Factor Xa, increase the expression of proinflammatory cytokines such as IL-8, IL-6, Monocyte-Chemotactic Protein-1, as well as Intercellular Adhesion Molecule-1 (ICAM-1) and Vascular Cell Adhesion Molecule-1 (VCAM-1). These molecules facilitate leukocyte adhesion and contribute to the recruitment of immune cells to the vascular endothelium. In this context, Nobiletti and colleagues highlighted the role of the *CCM1/KRIT1* gene in regulating neutrophil adhesion and motility. Neutrophil recruitment and the subsequent formation of neutrophil extracellular traps (*NETs)* have been observed in CCM lesions, indicating that immune cell activation is integral to CCM pathogenesis. *NET* formation, or *NET*osis, is a defense mechanism of neutrophils triggered by proinflammatory cytokines, activated platelets, and endothelial cells, contributing to vascular inflammation. Dömer et al. explored the broader immunological impact of NETs using human primary neutrophils in vitro. They demonstrated that *NETs* can mediate adaptative immunity by inducing the secretion of proinflammatory chemokines and cytokines or by presenting autoantigens to active B and T lymphocytes, further amplifying the inflammatory response. Additionally, *NET* formation relies on the generation of reactive oxygen species (ROS), a key pathological process in CCM development. ROS production promotes endothelial dysfunction, inflammation, and vascular remodeling, establishing a feedback loop that sustains the inflammatory and prothrombotic environment characteristic of CCM lesions [60,61,62,63].

**Figure 4:**
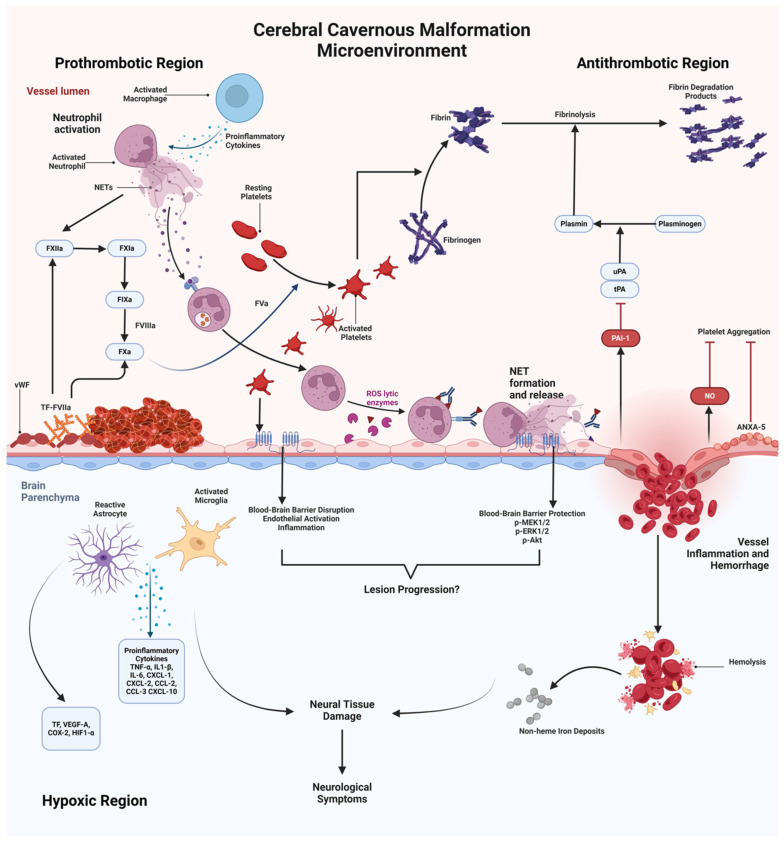
Pro- and Antithrombotic Processes occurring within the Cerebral Cavernous Malformation microenvironment. Graphical representation of both prothrombotic and antithrombotic regions within the Cerebral Cavernous microenvironment, where endothelial cells play a key role in regulating hemostasis, neuroinflammatory response and bleeding. Vascular endothelial cells interact with erythrocytes and diverse associated glycoproteins to regulate both coagulation and anticoagulation. Endothelial cells lacking CCM are unable to maintain an adequate hemostatic balance, leading to neuroinflammation and subsequent hypoxia within the brain parenchyma. This neuroinflammatory response, hypoxic state and hemorrhagic events are all hallmarks of Cerebral Cavernous Malformations, leading to further damage on neural tissue, which in turn manifests as neurological symptoms. ***NETs***, Neutrophil Extracellular Traps; **FXIIa**, Activated Factor XII; **FXIa**, Activated Factor XI; **FIXa**, Activated Factor IX; **FVIIIa**, Activated Factor VIII; **FXa**, Activated Factor X; **vWF**, Von Willebrand Factor; **TF**, Tissue Factor; **TF-FVIIa**, Tissue Factor-Activated Factor VII Complex; **FVa**, Activated Factor V; **ROS**, Reactive Oxygen Species; **uPA**, Urokinase Plasminogen Activator; **tPA**, Tissue Plasminogen Activator; **PAI-1**, Plasminogen Activator Inhibitor-1; **NO**, Nitric Oxide; **ANXA-5**, Annexin A-5 protein; **p-MEK1/2**, Phosphorylated Mitogen-Activated and Extracellular Signal-Regulated Kinase 1/2; **p-ERK1/2**, Phosphorylated Extracellular Signal-Regulated Kinase 1/2; **p-Akt**, Phosphorylated Protein Kinase B; **VEGF-A**, Vascular Endothelial Growth Factor-A; **COX-2**, Cyclooxygenase-2; **HIF1-α**, Hypoxia-Inducible Factor-1 alpha; **TNF-α**, Tumoral Necrosis Factor-alpha; **IL1-β**, Interleukin 1-betta; **IL-6**, Interleukin-6; **CXCL-1**, Chemokine (C-X-C motif) Ligand-1; **CXCL-2**, Chemokine (C-X-C motif) Ligand-2; **CXCL-10**, Chemokine (C-X-C motif) Ligand-10; **CCL-2**, Chemokine Ligand-2; **CCL-3**, Chemokine Ligand-3. Adapted from Globisch, M.A. et al. 2022 [57].

In summary, these findings suggest a disrupted balance between pro- and anti-thrombotic effectors within the CCM micro-environment, thus promoting the recruitment of proinflammatory cells and different cytokines, further affecting the vascular architecture and thus leading to the occurrence of hemorrhagic events [55,61] (Figure 4).

### 1.8. The Problem of Symptomatic Hemorrhage

Although the clinical course of CCM is highly unpredictable, it is generally considered benign, with an annual risk of clinically significant bleeding estimated at less than 0.5%. However, once a CCM lesion manifests with symptomatic hemorrhage, its natural course becomes considerably more serious. Studies have shown a re-bleeding rate of approximately 42% within the first five years if left untreated, often leading to cumulative disability [62,63,64,65,66].

Currently, CCM severity and progression are primarily assessed through MRI, but the high variability of the disease limits the accuracy of imaging in diagnosing, monitoring, and predicting its course. This underscores the urgent need for reliable biomarkers capable of distinguishing high-risk cases, guiding patient selection for invasive or novel treatments, and tracking disease progression and therapeutic response. The development of measurable plasma biomarkers would offer significant advantages for both patients and clinicians. Such clinical tests could enhance therapeutic decision-making, provide early indicators of disease activity, and predict clinical outcomes more effectively. Incorporating biomarker-based assessments into clinical practice could ultimately improve personalized treatment strategies and long-term management for individuals with CCM [64,67].

### 1.9. Plasmatic Molecular Biomarkers and Their Diagnostic and Prognostic Applications in Cerebral Cavernous Malformation Disease

After understanding the main molecular mechanisms involved in CCM pathogenesis, it is important to highlight the potential use of some of these molecules and cytokines as biomarkers. Several plasmatic proteins linked to these above-mentioned pathways are currently being investigated as potential biomarkers to reflect disease activity, severity, and progression in CCM [64].

### 1.10. Biomarkers: Context and Definitions

The usefulness of a plasmatic biomarker must be defined within a specific context of use to ensure its appropriate interpretation and application in medical practice. According to Girard et al., biomarkers can be classified into several categories based on their clinical utility (Table S1) [63].

The use of circulating plasma biomarkers holds significant potential for improving the surveillance and management of CCM disease by complementing MRI-derived diagnostic features. However, only a limited number of published studies have explored this area, resulting in a lack of consensus on which proteins might best predict the clinical course of CCM.

As the next step in this review, we aim to highlight the importance of circulating biomarkers in CCMs by summarizing the current state of knowledge. We will explore biomarkers proposed for the diagnosis, monitoring, and prognosis of both fCCM and sCCM.

## 2. Methods

### 2.1. Systematic Literature Review

We conducted a systematic literature review to identify all publications describing current knowledge regarding plasma biomarkers and their diagnostic, prognostic, and therapeutic applications in patients with CCM. The data query was performed between 15 January and 31 March 2024. This systematic review was conducted in accordance with the Preferred Reporting Items for Systematic Reviews and Meta-Analyses (PRISMA) 2020 guidelines [68]. To avoid database bias, searches were performed on PubMed, Embase, Google Scholar, and Cochrane Library using the Boolean operators “AND” and “OR”. The search string consisted of the following keywords: “cerebral cavernous malformation”, “cerebral cavernoma”, “familial cerebral cavernous malformation”, “brain cavernoma”, “biomarker”, “plasma biomarker”, and “circulating biomarker”. The complete electronic search strategy for all databases, including full Boolean search strings, is provided in the Supplementary Materials (Table S2).

All available results were exported to the Elsevier Reference Manager Mendeley (Elsevier; Amsterdam, the Netherlands) to check and suppress duplications. Titles and abstracts were independently screened by two reviewers (H.H.K. and E.G.). Full-text articles were independently assessed for eligibility by the same reviewers. Discrepancies were resolved through discussion, and consensus was reached in all cases. All articles with no relevance to plasma biomarkers in CCM patients were excluded. Eligible articles were entirely screened, as well as their reference lists for additional relevant articles, and added to the analysis. Inclusion criteria regarded articles, book chapters, clinical trials, meta-analyses, and randomized controlled trials conducted both in animal and human subjects investigating proposed molecular biomarkers and their potential diagnostic, prognostic, and therapeutic applications in patients with both cerebral and spinal cavernous malformations. Additional references were added by reviewing the reference lists of selected publications. The following information was extracted and summarized in tables: authors and date of publication, type of biomarker (i.e., plasma, tissular, or imaging), and the mechanisms of disease to which they are linked. Summarized data are presented in a descriptive manner.

### 2.2. Data Analysis

The literature search identified a total of 2899 records, including 1464 from PubMed, 703 from Embase, 711 from Google Scholar, and 21 from the Cochrane Library. After removal of duplicate records, 932 unique articles were screened based on titles and abstracts. Of these, 829 records were excluded because they did not meet the predefined inclusion criteria, including non-original articles (e.g., letters to the editor or secondary publications), studies without relevant clinical or molecular data, experimental studies lacking translational relevance, and investigations focusing on non-CNS cavernous hemangiomas or other unrelated vascular malformations.

**Figure 5:**
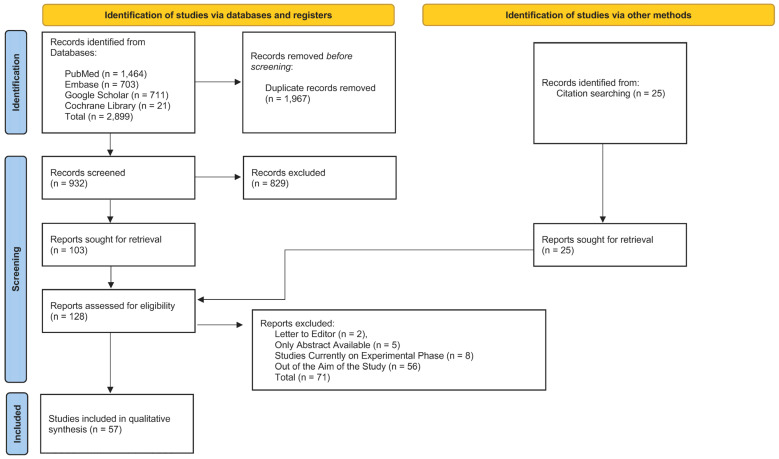
PRISMA 2020 flow diagram of the study selection process, adapted from Page et al., 2021 [68].

Full-text reports were sought for 103 articles identified through database searching. In addition, 25 records were identified through citation searching and assessed directly at the full-text level. In total, 128 articles were assessed for eligibility. Following full-text evaluation, 71 articles were excluded for predefined reasons, and 57 studies were ultimately included in the qualitative synthesis. The study selection process is summarized in a PRISMA 2020 flow diagram (Figure 5). Details are provided in Table 1.

## 3. Results and Discussion

### 3.1. Literature Review

A total of 57 studies published between 1984 and 2024 were reviewed. Most studies originated from North American institutions (70.17%), followed by European centers (19.29%), with fewer contributions from Asia (7.01%) and South America (3.50%). Across these studies, 62 different biomarkers were identified. Regarding biomarker types, 45 studies (78.94%) described 55 circulating plasma molecules proposed as potential biomarkers in both familial and sporadic cases. Eight studies (14.03%) focused on two specific MRI sequences with potential diagnostic and prognostic applications as “neuroimaging biomarkers”. Three studies (5.26%) explored at least two molecular biomarkers related to gut microbiome composition and its clinical relevance in CCM disease. Lastly, one study (1.75%) described a set of tissue biomarkers identified in endothelial cells of CCM lesions.

Based on the mechanisms involved in CCM pathology, 60 biomarkers (96.77%) identified in the reviewed studies are associated with processes such as inflammation, endothelial angiogenesis, and immune response regulation. The remaining two biomarkers (3.22%) are linked to the gut micro-environment, reflecting the emerging recognition of the gut–brain axis in CCM disease.

### 3.2. Identification and Validation of Circulating Plasma Biomarkers

Biomarkers play a critical role in predicting, screening, diagnosing, and stratifying risk across a wide number of diseases. They can be cellular, histological, molecular, physiological, or radiographic characteristics, used either independently or as part of a “disease-related panel” targeting multiple biomarkers to increase diagnostic sensitivity and specificity. Among these, circulating plasma biomarkers are particularly advantageous due to their low cost, feasibility, and acceptability for diagnostic and prognostic applications. In fields like oncology, biomarkers have been extensively studied to assess correlations between drug treatments and clinical outcomes, predict patient response to therapies, and monitor disease progression or recurrence. A key hallmark of some tumors is the localized increase in angiogenic signals, which supports the formation of new vasculature essential for tumor growth. Similar dysregulated angiogenic and inflammatory processes occur in vascular malformation diseases, including CCM. In this context, identifying specific biomarkers could be instrumental in detecting individuals at high risk of developing neurological deficits. This risk is closely linked to lesion expansion and hemorrhage, emphasizing the potential clinical utility of biomarker-based monitoring in guiding clinical decision-making [97,102,105].

### 3.3. The Problem with Symptomatic Hemorrhage

Although CCMs are generally considered benign, they pose a lifelong risk of serious neurologic complications, including stroke, seizures, and disability due to lesion-related hemorrhage. The annual risk of clinically significant hemorrhage in CCM patients is estimated at 0.5 to 2.4%, with a cumulative risk of 20% over five years. This risk is even higher in patients with SCMs, reaching 40% within the same period. Once an initial symptomatic hemorrhage occurs, the risk of rebleeding increases substantially, reaching 30% to 42% in CCM patients and up to 55% in SCM patients within five years. This progression underscores the importance of early detection and monitoring in affected individuals. The term Cavernous Angioma with Symptomatic Hemorrhage (CASH) has been introduced to describe a clinically significant event characterized by evidence of new lesional bleeding or hemorrhagic lesion growth greater than 3 mm on MRI, accompanied by corresponding neurological symptoms. The CASH definition serves as a critical diagnostic and prognostic marker in clinical practice, helping guide therapeutic decisions and long-term management strategies for patients with CCM and SCM [63,66,100,105,113].

Recent advances in understanding the biological processes underlying CCM disease have opened new avenues for characterizing molecular signatures associated with CASH lesions, offering potential tools for predicting future bleeding events. In this context, Girard et al. identified a “proinflammatory cluster” of plasma cytokines, including vitamin D and non-HDL cholesterol levels, correlating with chronic CCM disease severity. The same group further demonstrated the prognostic value of elevated plasma levels of four inflammatory and angiogenic biomarkers—sCD14, IL-1 β, VEGF, and ROBO4—in predicting symptomatic hemorrhage within the first year after an initial bleeding event. Using traditional statistical analyses and machine learning models, they showed that a weighted combination of these biomarkers achieved an 86% sensitivity and 88% specificity in forecasting future hemorrhages. Additionally, they proposed a diagnostic biomarker panel composed of sCD14, CRP, IL-10, and VEGF, which demonstrated a sensitivity of 80% and specificity of 77% [63,100].

Lyne et al. examined the differences between the diagnostic and prognostic biomarkers, identifying significant relationships among five of the six studied molecules. This finding suggests the presence of both shared and distinct components of inflammatory and angiogenic pathways involved in the progression of CCM following an initial hemorrhagic event and contributing to the risk of future rebleeding. Further transcriptome analysis of CASH-related molecules revealed a set of dysregulated genes linked to these biomarkers [100] (Table 2).

Some key elements of neuroinflammation are well documented, including the role of IL-10 in modulating inflammatory responses and promoting recovery. IL-10 plays a central role in the metabolic reprogramming of macrophages by inhibiting LPS uptake, thereby reducing the inflammatory response. In the context of cerebral injuries, IL-10 contributes to resolving inflammation by promoting M2 macrophage activation through Signal Transducer and Activator of Transcription 3 (STAT3) pathway. This activation triggers anti-inflammatory and tissue-repair processes, enhancing neuroprotection and supporting the restoration of brain homeostasis. By limiting excessive inflammation and facilitating tissue repair, IL-10 serves as a critical regulator in the neuroinflammatory landscape [100,114,115,116].

C-Reactive Protein (CRP) is a well-established biomarker of systemic inflammation and is routinely used to monitor the acute phase of inflammatory responses. Its hepatic synthesis is stimulated by cytokines such as IL-6, causing plasma levels to rise up to 500-fold within 24–48 h of an acute inflammatory event. CRP plays a key role in the immune response by activating complement proteins, enhancing pathogen clearance, and promoting immune cell recruitment. CRP has garnered significant attention as a cardiovascular disease risk marker due to its consistent association with both subclinical and clinically evident vascular disease progression across diverse populations. Mineo et al. demonstrated that CRP contributes to endothelial dysfunction by antagonizing endothelial nitric oxide synthase (eNOS) activation. This occurs through changes in eNOS phosphorylation mediated by Protein Phosphatase 2A (PP2A), disrupting nitric oxide (NO) production. Importantly, this CRP-induced eNOS antagonism has been observed at CRP levels previously linked to increased cardiovascular disease risk. In CCM patients, elevated CRP plasma levels can persist for up to a year following a symptomatic hemorrhagic event. This prolonged elevation suggests the presence of ongoing inflammatory processes that sustain a proinflammatory microenvironment even after the acute phase has resolved. Additionally, CRP-induced NO suppression promotes monocyte adhesion through its interaction with the CRP-related receptor FCGR2B. This mechanism may contribute to a positive feedback loop that perpetuates inflammatory signaling, further supporting CRP’s potential role as a biomarker for chronic vascular inflammation and lesion progression in CCM disease [100,117,118].

Both sCD14 and VEGF were identified as key biomarkers in both diagnostic and prognostic CASH profiles described by Lyne et al. CD14 was initially characterized as a membrane-bound glycosylphosphatidylinositol-anchored protein and a cell surface differentiation marker found on monocytes, macrophages, dendritic cells, and neutrophils. It functions as a receptor for bacterial LPS in cooperation with TLR-4 and TLR-2, mediating bacterial phagocytosis by facilitating the binding of bacterial ligands to receptors on phagocytes. CD14 also exists in two soluble forms with different molecular masses. This is particularly relevant because sCD14 enables cells lacking membrane-bound CD14, such as epithelial and endothelial cells, to respond to LPS. In murine models, brain endothelial cells expressing CD14 receptors were shown to mediate LPS-induced CCM lesion development. Tang et al. reported that functional gene variants enhancing membrane-anchored CD14 expression were associated with a higher lesion count in fCCM patients sharing a *Q455X* mutation. Interestingly, lower levels of sCD14, rather than higher, were found to be significant in both the diagnostic and prognostic CASH biomarker profiles. This finding contrasts with the expected pro-inflammatory role of CD14. Higher sCD14 levels may have an anti-inflammatory effect by competitively inhibiting LPS-mediated responses, possibly preventing excessive activation of TLR-mediated pathways. This suggests that in CCM, reduced sCD14 levels might fail to counterbalance inflammatory signaling, thereby promoting a pro-inflammatory environment that supports lesion progression. The precise regulatory mechanisms driving sCD14 downregulation remain unclear but likely involve complex interactions within the inflammatory microenvironment characteristic of CCM pathology [100,119,120].

VEGF has been extensively studied as a central factor in CCM pathogenesis, playing a critical role in both lesion formation and progression. As a pro-mitotic and pro-migratory growth factor, VEGF is intimately involved in endothelial activation, angiogenesis, and the regulation of vascular permeability. Numerous studies have shown that VEGF and its receptors are overexpressed in CCM lesions compared to normal brain vessels, emphasizing its importance in promoting endothelial dysfunction and vascular remodeling. Interestingly, in CASH patients, lower plasma levels of VEGF have been observed in those who experienced a hemorrhagic event within the previous year and in those at risk of bleeding in the following year. This finding appears counterintuitive, given VEGF’s established role in angiogenesis and endothelial activation. However, it suggests that aberrant VEGF signaling rather than absolute VEGF deficiency may underlie the persistent vascular instability seen in CCM patients. One proposed mechanism is that chronic VEGF dysregulation may impair normal vascular repair processes while promoting abnormal angiogenic signaling, contributing to the development of leaky, structurally weak vessels prone to hemorrhage. This disruption in VEGF pathway signaling could create a state of sustained endothelial vulnerability, predisposing CCM lesions to recurrent bleeding despite relatively low circulating VEGF levels [54,100,121].

IL-1 β is a well-characterized proinflammatory cytokine involved in mediating inflammation-induced angiogenic responses. It indirectly promotes the synthesis of various pro-angiogenic factors while facilitating endothelial cell migration. Additionally, IL-1β can increase vascular endothelial permeability by enhancing leukocyte adhesion and promoting transmigration across the endothelial barrier, contributing to vascular inflammation and tissue remodeling. Girard et al. found that higher IL-1β levels, coupled with lower IL-6 levels, were associated with increased clinical activity in CCM patients. This observation supports the hypothesis that inflammatory-driven endothelial permeability plays a central role in lesion progression, leading to either lesional expansion or symptomatic hemorrhage. The dual role of IL-1β as both an angiogenic mediator and an inducer of vascular permeability highlights its significance in the inflammatory microenvironment characteristic of CCM. Its influence on leukocyte recruitment, endothelial activation, and vessel destabilization underscores its potential as a biomarker for disease activity and a therapeutic target aimed at controlling inflammation-driven vascular instability in CCM patients.

Finally, the Roundabout (Robo) family of proteins functions as guidance receptors with well-established roles within the CNS. Among them, ROBO4 is expressed specifically by vascular endothelial cells, where it acts as an endogenous inhibitor of VEGF signaling. In mouse models of retinal and choroidal vascular diseases, activation of ROBO4 by its ligand Slit2 reduced both angiogenesis and vascular leakage, while deletion of ROBO4 exacerbated these pathologic processes. Girard et al. found persistently elevated plasma levels of ROBO4 in CCM patients who experienced clinical lesional activity within the year following an initial blood sample. This increase in ROBO4 may reflect a compensatory metabolic response aimed at counteracting the heightened endothelial permeability driven by underlying proinflammatory processes. By stabilizing the endothelium and reducing vascular leakiness, elevated ROBO4 levels could represent a homeostatic mechanism attempting to mitigate disease progression. The association between increased ROBO4 levels and active CCM lesions supports its potential role as a prognostic biomarker. Its regulatory effects on vascular integrity and angiogenesis suggest that ROBO4 might serve as both a marker of disease activity and a possible therapeutic target for limiting CCM growth and reducing hemorrhage risk [82,95,122].

### 3.4. Gut Microbiome-Related Studies

As previously mentioned, the “Gut–Brain Axis” has emerged as one of the multiple factors contributing to CCM genesis and progression. This concept stems from murine studies showing that an increased abundance of GNB in the gut microbiota correlated with heightened LPS-induced TLR4 signaling, a pathway implicated in CCM development. Moreover, both mouse and human studies have suggested that specific polymorphisms affecting TLR expression may influence the clinical course of CCM disease. Building on this hypothesis, Polster et al. investigated whether CCM patients have a distinctive gut microbiome compared to healthy individuals. Their analysis revealed an upregulation of LPS-synthesis-related genes in CCM patients, suggesting that gut-derived LPS may play a critical role in the disease’s natural history. Using metagenomic shotgun sequencing and taxonomic identification of genome clusters, they identified three key bacterial species with diagnostic significance: The Gram-negative *Odoribacter splachnicus*, which was significantly increased, and the Gram-positive *Faecalibacterium prausnitzii* and *Bifidobacterium adolescentis*, which were significantly decreased. Comparing these three species in a weighted diagnostic model yielded a sensitivity of 92% and specificity of 67% for diagnosing CCM [49,91].

Patients presenting with CASH may exhibit a distinct gut microbiome profile. Multivariate analyses have identified six bacterial taxa associated with CASH status, including *Faecalibacterium prausnitzii*, *Oscillobacter*, *Lactobacillus rhamnosus*, *Enterobacter cloacae*, *Odoribacter laneus*, and *Bacteoides cullulosiltycus*. A weighted combination of these taxa generated a ROC curve that moderately distinguished CASH from non-CASH patients. These findings reinforce the concept of a gut–brain axis in CCM pathophysiology and highlight the potential of microbiome-based biomarkers for risk stratification and personalized disease management [91].

Both *F. prausnitzii* and *B. adolescentis* are Gram-positive bacterial species recognized for their protective role against gut inflammation. They contribute to maintaining intestinal mucosal homeostasis by producing anti-inflammatory metabolites such as butyrate, which supports gut barrier integrity and modulates immune responses. A reduction in these beneficial species, as observed in CCM patients, could compromise gut barrier function, leading to increased intestinal permeability and promoting systemic inflammation. This dysregulation may exacerbate CCM progression by enhancing the gut–brain inflammatory axis. In contrast, Gram-negative species like *O. splanchnicus*, which are more abundant in CCM patients, can induce gut inflammation through LPS production. Notably, *O. splanchnicus* has also been associated with neuroinflammatory conditions like Alzheimer’s disease, further supporting the idea that gut dysbiosis contributes to neurological disorders through immune and inflammatory pathways [123,124,125,126,127].

Despite significant progress in identifying a potential link between the distinctive gut microbiome and plasma proteins implicated in CCM disease activity, the association remains limited and requires further validation. Current evidence suggests that the relationship between these factors may be complementary rather than directly causative. The gut microbiome likely influences systemic inflammation, while plasma proteins reflect ongoing vascular and immune processes within CCM lesions. This emerging understanding highlights an open area of opportunity for future research aimed at validating combinations of gut-derived microbial signatures and circulating plasma biomarkers as potential prognostic tools. Such integrated biomarker panels could improve risk assessment, monitor disease progression, and guide personalized therapeutic strategies in CCM disease management. Expanding studies in this direction could ultimately lead to a more comprehensive understanding of CCM pathophysiology and better clinical outcomes for affected patients.

### 3.5. Magnetic Resonance Imaging Modalities as In Vivo Biomarkers

According to current consensus recommendations for the clinical management of CCM, MRI remains the gold standard for detecting and characterizing CCMs due to its near-perfect sensitivity and high specificity. However, standardized follow-up protocols have yet to be established and are influenced by various factors, including the patient’s medical insurance coverage, the treating physician’s clinical judgment, patient preferences, and the presence and severity of neurological symptoms. In this context, advanced imaging techniques such as Quantitative Susceptibility Mapping (QSM) and Dynamic Contrast-Enhanced Quantitative Perfusion (DCEQP) have been proposed as promising MRI-based biomarkers for monitoring CCM progression. These techniques are currently being evaluated in clinical trials to assess their utility in disease surveillance and risk prediction. QSM, in particular, has gained attention as a follow-up biomarker across a range of neurologic disorders characterized by iron deposition, including cerebral microbleeds, multiple sclerosis, various brain tumors, and neurodegenerative diseases [110,128,129,130,131].

Mikati et al. explored the potential of advanced MRI techniques, including QSM and DCEQP, as biomarkers for assessing CCM disease activity and treatment response. Their study demonstrated a positive correlation between the mean QSM susceptibility and the mean permeability of CCM lesions, particularly in fCCM, suggesting that lesions with greater iron deposition also exhibit increased vascular permeability. Additionally, they identified a significant positive correlation between QSM-measured susceptibility and Cerebral Blood Volume (CBV) in CCM lesions, indicating that higher iron accumulation is associated with increased lesion vascularity. However, no correlation was observed between QSM susceptibility and Cerebral Blood Flow (CBF), suggesting that lesion-specific vascular changes primarily involve blood volume and vessel permeability rather than overall cerebral perfusion. These findings support the potential of QSM and DCEQP as imaging biomarkers for quantitatively assessing the biological behavior of CCM lesions. Their ability to reflect key pathophysiological features such as iron deposition, vascular permeability, and lesion vascularity underscores their value in monitoring disease progression and evaluating treatment efficacy in CCM patients [82,107,108,111,112].

The transcriptome profile of CASH lesions reveals differentially expressed genes encoding angiogenic and inflammatory proteins that regulate vascular perfusion and permeability. A notable example is VEGF, a key regulator of angiogenesis, tissue perfusion, and blood–brain barrier permeability, particularly during the hypoperfusion state following a hemorrhagic stroke. Sone et al. investigated the diagnostic efficacy of DCEQP compared to circulating plasma biomarkers identified by Girard and colleagues. They concluded that combining perfusion-based imaging biomarkers with circulating plasma proteins could enhance diagnostic accuracy [106,132,133].

## 4. Conclusions

We conducted a comprehensive review of the current understanding of CCM physiopathology, focusing on recent discoveries in molecular pathways involved in CCM genesis and disease progression. Additionally, we provide an extensive systematic literature review addressing ongoing efforts to identify circulating plasma molecules as potential biomarkers for risk stratification and patient monitoring in various clinical settings. Our review emphasizes the relevance of these biomarkers in improving diagnostic accuracy, predicting disease progression, and guiding personalized treatment approaches. From a molecular medicine standpoint, the integration of circulating biomarkers with mechanistic insights may enable biomarker-driven risk stratification, disease monitoring, and the development of targeted therapeutic strategies in CCM.

We hope that this synthesis of current knowledge will serve as a foundation for future clinical trials aimed at evaluating the feasibility, precision, and reproducibility of circulating plasma biomarkers as indicators of CCM disease activity and clinical outcomes.

## Figures and Tables

**Table 1:** Candidate Circulating Plasma and Tissular Biomarkers Included in our Systematic Review.

Biomarker	Mechanisms of Disease	Rationale	Reference
**25-OH Vitamin D**	Inflammation process, autophagy, immune response	Lower plasmatic levels of Vitamin D have been observed in CCM patients with a more aggressive disease course. Also, peripheral plasma vitamin D has been shown to reflect the severity of CCM disease.	Girard et al. 2016 [69] Gibson et al. 2015 [70] Venugopal et al. 2022 [71] Flemming et al. 2020 [72]
***CCL2/MCP-1***	Inflammation	Increased CCL2/MCP-1 induces Blood–Brain Barrier permeability and activates the Rho/ROCK pathway.	Retta et al. 2016 [3] Keep et al. 2014 [73] Luissint et al. 2012 [74]
**C-reactive protein (CRP)**	Inflammation process, autophagy, immune response	CCM3 polymorphism is associated with increased CRP plasma levels. CRP increases Blood–Brain-Barrier permeability.	Gao et al. 2018 [75] Hsuchou et al. 2012 [76] López-Ramírez et al. 2019 [53]
**Endoglin**	Inflammation process, autophagy, immune response	Soluble endoglin induces cerebral endothelium remodeling and may promote sporadic AVM formation. Endoglin gene loss is an identified cause of hereditary hemorrhagic telangiectasia.	Chen et al. 2009 [77] McAllister et al. 1994 [78]
**Thrombomodulin/Thrombospondin 1 (TSP1)**	Coagulation domains and Thrombin–endothelial interactions, angiogenesis	KRIT1 gene inactivation causes an upregulation of KLF2/4 factors which in turn inhibits Thrombomodulin/Thrombospondin 1 expression, creating a permissive microenvironment for angiogenic factors.	López-Ramírez et al. 2017 [79] DiStefano et al. 2020 [54]
**Platelet factor-4 (CXCL-4)**	Inflammation, angiogenesis	It has been observed that high blood levels of Platelet factor-4 is associated with inflammatory, and matrix mechano-transduction processes involved in CCM formation in familial cases.	Lazzaroni et al. 2024 [67]
**Granulocyte–macrophage Colony-stimulating factor (GM-CSF)**	Inflammation process, autophagy, immune response	Asymptomatic CCM patients may show elevated plasma levels of GM-CSF compared to symptomatic patients.	Chehuen-Bicalho et al. 2021 [80]
**Galectin-3** **(Gal-3)**	Inflammatory response	Studies in cellular and animal models have shown that Gal.3 expression levels are inversely correlated with those of KRIT1, one of the major causative genes for CCM disease, implying a functional relationship.	Kar et al. 2024 [81]
**Cholic/Glycocholic Acid**	Inflammation, angiogenesis	It has been shown that glycocholic acid inhibits VEGF-induced angiogenesis in choroidal endothelial cells and protects against oxidative damage. Also, it has been described that CCM patients may express different plasma concentration in comparison to healthy subjects	Srinath et al. 2023 [64]
**Arachidonic/Linolenic Acid**	Inflammation process, autophagy, immune response	Patients presenting with symptomatic hemorrhage showed greater plasma levels of both metabolites. It is proposed that LPS, in addition to its role in TLR-4 signaling and MAPK/MEKK3/ERK3 upregulation, indices COX-1/COX-2 expression, possibly explaining the increased blood levels seen in those patients.	
**Seric Bilirubin**	Inflammation process, autophagy, immune response	CCM patients with an aggressive course show lower levels of bilirubin, suggesting a possible link between reduced bilirubin antioxidant effect and cumulative activity of CCM disease during a patient’s life.	
**Interferon gamma (IFN γ)**	Endothelial permeability inflammation	Increased plasmatic levels of IFN γ in CCM patients is associated with an aggressive clinical course of disease. IFN γ increases endothelial permeability and induces cytoskeletal derangements.	Girard et al. 2018 [82] Ng et al. 2015 [83]
**Interleukins:**			
**IL-1 β**	Inflammation, immune response, angiogenesis	Lower plasmatic levels of IL-6 and elevated levels of IL-1 β in CCM patients who experienced clinical lesional activity within the first year following the initial blood sample.	Girard et al. 2018 [62] Girard et al. 2018 [82] Pawlikowska et al. 2004 [84] Jauhiainen et al. 2022 [85] Chehuen-Bicalho et al. 2021 [80]
**IL-2**			
**IL-6**		Increased plasmatic levels of IL-2 and IL-1 β is associated with an aggressive clinical course.	
**IL-8**			
**IL-10**		IL-27 can promote Th1 differentiation and consequent activation of the inflammatory response. It has been observed that asymptomatic CCM patients tend to show increased plasma levels of IL-27.	
**IL-27**			
**Leukocyte-EC Adhesion:**			
**Vascular Cell Adhesion Molecule 1 (VCAM-1)**	Inflammation, endothelial permeability	Increased expression of ICAM-1/VCAM-1 on endothelial cells is a biomarker of inflammation.	Lampugnani et al. 2018 [86] Robinson et al. 1995 [87] Jauhiainen et al. 2024 [88]
**Intercellular Adhesion Molecule 1 (ICAM-1)/Cluster of Differentiation 54 (CD54)**		CCM lesions tend to show a lower expression of ICAM-1/VCAM-1.	
**Lipoproteins:**			
**High-Density Lipoprotein (HDL)** **Non-HDL Cholesterol** **Low-Density Lipoprotein (LDL)** **Triglycerides**	Inflammation	Simvastatin downregulates Rho/ROCK activity in CCM lesions on mature mice. Lower levels of non-HDL cholesterol are associated with a more aggressive clinical presentation. Simvastatin rescues CCM phenotype in preclinical studies in mice.	Shenkar et al. 2017 [89] Girard et al. 2016 [69] Whitehead et al. 2009 [22]
**Extracellular Matrix Proteins & Gut Microbiome Proteins:**			
**MMP-2 MMP-9**	Extracellular matrix remodeling, endothelial permeability	Plasma levels of MMP-2 and MMP-9 were respectively higher and lower in CCM patients with previous seizure activity. Higher expression levels of MMP-2 and MMP-9 may explain cases of CCM with subclinical hemorrhage.	Girard et al. 2018 [62] Bicer et al. 2010 [90]
**ADAMTS4**	MAPK signaling, PI3K-mTOR signaling, microbiome mechanisms, inflammatory process.	There is evidence of a strong synergy between loss of KRIT1 and ADAMTS4/5 gain in brain endothelial cells during early stages of CCM lesion formation.	Hong et al. 2020 [40]
**ADAMTS5**			
**Toll-like Receptor 4 (TLR-4)**		Studies demonstrate that genetic changes associated with altered TLR-4 and CD-14 expression result in coordinate changes in CCM lesion formation in both humans and mice.	Tang et al. 2017 [49]
**LPB/LPS**		Studies shown that blood-borne GNB and LPS are strong drivers of CCM formation in mice models through activation of TLR-4/MEKK3/KLF2-4 signaling pathways.	Polster et al. 2020 [91]
**Mucin-2 Glycoprotein (MUC-2)**		MUC-2 is a glycoprotein secreted by goblet cells and is the primary constituent of the colonic mucosal barrier. Loss of PDCD10 on intestinal endothelial cells is associated with disruption of the colonic mucosal barrier.	Tang et al. 2019 [45] Bergstrom et al. 2010 [92] Chassaing et al. 2012 [93]
**Fecal Lipocalin-2 (LCN-2)**		Fecal Lipocalin-2 is a secreted inflammatory response protein that has been shown to be a sensitive and dynamic marker of colitis in murine models.	
**Roundabout 4. (ROBO-4)**	Angiogenesis, endothelial permeability	Greater plasma levels of ROBO-4 in CCM subjects who experienced clinical lesional activity within the first year following an initial blood sample.	Girard et al. 2018 [62] Wüstehube et al. 2010 [94] Jones et al. 2008 [95]
		CCM1 gene is associated with a downregulation of ROBO-4 gene expression.	
		ROBO-4 gen inhibits endothelial hyper-permeability and abnormal angiogenesis.	
**Soluble cluster of differentiation 14 (sCD14)**	Inflammation	Lower plasma levels of sCD14 in CCM subjects who experienced clinical lesional activity within the first year following an initial blood sample. CD14 polymorphisms coding for the anchored membrane are associated with higher CCM lesion burden in familial-CCM cases. CD14 polymorphisms are associated with an increased susceptibility for high CCM lesions burden.	Girard et al. 2018 [62] Tang et al. 2017 [49] Choquet et al. 2014 [96]
**Transforming Growth Factor Beta Receptor-3 (TGF-βR3)**	Angiogenesis, endothelial permeability	TGF-βR3 belongs to one of the three types of TGF-β receptors, which has been implicated in the pathology of CCM.	Wetzel-Strong et al. 2021 [97]
**Tumor Necrosis Factors:**			
**TNF-α** **TNF-RI**	Inflammation	Increased TNF-α plasma levels in CCM patients is associated with an aggressive clinical course. Homozygosity for the TNF-α 308 GG genotype is associated with a greater risk of intracerebral hemorrhage in AVMs.	Girard et al. 2018 [62] Pawlikowska et al. 2004 [84] Cunha et al. 2017 [98] Jung et al. 2003 [99]
**Vascular Endothelial Growth Factor (VEGF)**	Angiogenesis, endothelial permeability	Lower plasma levels of VEGF in CCM subjects who experienced clinical lesional activity within the first year following an initial blood sample. VEGF is associated with vasculogenesis and endothelial permeability. Also, VEGF expression has dynamic changes during the clinical course of CCM disease.	
**Micro-Ribonucleic Acids:**			
***hsa-miR-363-3p*** ***hsa-miR-486-5p*** ***hsa-miR15a-5p*** ***hsa-miR-25-3p*** ***hsa-miR106b-3p*** ***hsa-miR-16-2-3p*** ***hsa-miR-183-5p*** ***hsa-miR-16-5p*** ***hsa-miR185-5p*** ***hsa-miR-501-3p*** ***hsa-miR-181a-5p*** ***hsa-miR.532-5p*** ***hsa-miR-7641-2-3p_novel***	Inflammation, angiogenesis	Micro-Ribonucleic Acids isolated from diverse body fluids, including plasma, have been shown to rescue endothelial phenotype and to inhibit vasculogenesis through downregulation of pro-inflammatory interleukins.	Girard et al. 2021 [63] Lyne et al. 2019 [100] Venugopal et al. 2022 [71] Florian et al. 2021 [101]
**Coagulation Signaling-Related Factors:**			
**Plasma Kallikrein (Pka)**	Inflammation, immune response, angiogenesis	Implicated in blood coagulation, fibrinolysis, hemostasis, and inflammatory response. Low plasmatic levels of Kallikrein leads to vascular bleeding and has been implicated in hereditary angioedema and hemorrhagic stroke in Hispanic population.	Croft et al. 2024 [102]
***Serpin Family F Member 1/2 (SERPINF1/2)***		Plays critical roles in vascular angiogenesis and has been implicated in retinal vascular leakage and hemorrhagic stroke.	
**Peptidoglycan Recognition Protein-2 (PGLYRP2)**		Has a role in immunomodulation and innate immunity, and has been associated with hemorrhagic stroke.	
***Adenomatous Polyposis Coli Gene (Apc)***		Has a critical role in development, negatively regulating Wnt signaling, and may be involved in angiogenesis.	
**Retinol Binding Protein 4 (RBP-4)**		There is evidence of a strong link between RBP-4 and the severity of diverse cardiovascular disorders, also associated with hemorrhagic stroke.	
**Genes Expressing Single-Nucleotide Polymorphisms (SNPs):**			
**Protein Tyrosine Phosphatase Non-Receptor Type 2 (PTPN2)**	Inflammation, immune response, angiogenesis	SNPs in the PTPN2 in combination with other variants have been shown to possibly increase the susceptibility of chronic inflammatory disorders. Also, patients who inherit the PTPN2 rs72872125 genotype have high levels of IL-10 when compared with controls.	Galvao et al. 2024 [103]
**Vitamin D Receptor (VDR)**		Vitamin D3 decreases CCM lesion burden through inhibiting ROCK activity in murine models. Some SNPs in genes involved in Vitamin D signaling have been reported to have association with vitamin D deficiency.	
**Low Affinity Immunoglobulin Gamma Fc Region Receptor II-a (FCGR2A)**		Mediates changes in endothelial function and inflammatory response, thus being capable of increasing the expression of ICAM-1 and E-Selectin in human umbilical vein endothelial cells.	
**Small Nucleolar RNAs (snoRNAs) on CCM Tissue:**			
***SNORD115-32***	Angiogenesis and vascular homeostasis	Recent studies have provided evidence for snoRNAs’ role in CCM pathogenesis, concluding that the robust down-regulation of SNORD115-32 and SNORD114-22 may be of biological and functional relevance for patients suffering from CCM within the brainstem.	Kar et al. 2018 [104]
***SNORD114-22***			
***SNORD113-3***			
**Candidate Imaging Biomarkers:**			
**Dynamic Contrast-Enhanced Quantitative Perfusion (DCEQP)**	Rho/ROCK-mediated vascular hyperpermeability	Allows assessment of brain vascular permeability in CCM subjects. Also, greater permeability in white matter far from lesions has been observed in familial CCM cases than in sporadic cases, suggesting a correlation with a more aggressive CCM disease. Increased permeability would reflect current on-going rate of endothelial leaking.	Kim et al. 2021 [105] Sone et al. 2021 [106] Girard et al. 2017 [107] Mikati et al. 2015 [108]
**Quantitative Susceptibility Mapping (QSM)**		A method developed to measure the magnetic susceptibility of brain tissue, an intrinsic biophysical property of the tissue that is directly proportional to the local iron content. In CCM patients it would reflect the integral or historical accumulation of leaking through iron deposition.	Hage et al. 2023 [109] Zeineddine et al. 2018 [110] Girard et al. 2017 [107] Tan et al. 2016 [111] Mikati et al. 2014 [112]

CCL2/MCP-1, Monocyte Chemotactic Protein 1; CRP, C-Reactive Protein; TSP1, Thrombospondin 1; CXCL-4, Platelet Factor 4 (C-X-C motif ligand 4); GM-CSF, Granulocyte-macrophage Colony-stimulating Factor; Gal-3, Galectin 3; IFN γ, Interferon Gamma; IL-1β, Interleukin-1β; IL-2, Interleukin-2; IL-6, Interleukin-6; IL-8, Interleukin-8; IL-10, Interleukin-10; IL-27, Interleukin-27; VCAM-1, Vascular Cell Adhesion Mollecule-1; ICAM-1, Intercellular Adhesion Mollecule-1; CD54, Cluster of Differentiation-54; HDL, High-Density Lipoprotein; LDL, Low-Density Lipoprotein; MMP-2, Matrix Metalloproteinase-2; MMP-9, Matrix Metalloproteinase-9; ADAMTS4, ADAM metallopeptidase with thrombospondin type 1 motif 4; ADAMTS5, ADAM metallopeptidase with thrombospondin type 1 motif 5; TLR-4, Toll-Like Receptor-4; LPB, Lipopolysaccharide Binding Protein; LPS, Lipopolysaccharide; MUC-2, Mucin-2 Glycoprotein; LCN-2, Fecal Licopalin-2; ROBO-4, Roundabout homolog-4; sCD14, Soluble cluster of differentiation 14; TGF-Rβ3, Transforming Growth Factor Beta Receptor-3; TNF-α, Tumoral Necrosis Factor-alpha; TNF-RI, Tumoral Necrosis Factor Receptor-1; VEGF, Vascular Endothelial Growth Factor; Pka, Plasma Kallikrein; *SERPIN-1*, *Serpin Family F Member-1*; *SERPIN-2*, *Serpin Family F Member-2*; PGLYRP2, Peptidoglycan Recognition Protein-2; Apc, *Adenomatous Polyposis Coli Gene*; RBP-4, Retinol Binding Protein-4; PTPN2, Protein Tyrosine Phosphatase Non-Receptor Type 2; VDR, Vitamin D Receptor; FCGR2A, Low Affinity Immunoglobulin Gamma Fc Region Receptor II-a; CCM, Cerebral Cavernous Malformation; Rho/ROCK, Ras homolog gene family member/Rho-associated protein kinase; AVM, Arteriovenous Malformation; KRIT1, Krev Interaction Trapped Protein 1; CCM3, Cerebral Cavernous Malformation 3 Protein; KLF2/4, Kruppel-like Factor 2/4; MAPK, mitogen-activated protein kinase; MEKK3, dual specificity mitogen-activated protein kinase kinase 3; ERK3, Extracellular signal-Regulated Kinase 3; COX-1, Cyclooxygenase-1; COX-2, Cyclooxygenase-2. DCEQP, Dynamic Contrast-enhanced Quantitative Perfusion; **QSM**, Quantitative Susceptibility Mapping; Adapted from Snellings et al. 2021 [1] and Girard et al. 2021 [63].

**Table 2:** Dysregulated genes related to biomarkers in the CASH transcriptome.

Gene	Associated Plasma Biomarker	Related Disease Mechanisms	Biomarker’s Utility
**IL10RA**	IL-10	Inflammation	Diagnostic
**CD14**	sCD14	Inflammation	Diagnostic/Prognostic
**VEGFA**	VEGF	Angiogenesis/Endothelial permeability	Diagnostic/Prognostic
**FLT1**	VEGF	Angiogenesis/Endothelial permeability	Diagnostic/Prognostic
**FCGR2B**	CRP	Inflammation	Diagnostic
**CASP1**	IL-1 β	Inflammation	Prognostic
**IL1R2**	IL-1 β	Inflammation	Prognostic
**ROBO4**	ROBO4	Angiogenesis/Endothelial permeability	Prognostic

**IL10RA**, Interleukin 10 Receptor subunit alpha; **IL-10**, Interleukin 10; **CD14**, Cluster of Differentiation 14; **sCD14**, Plasmatic/Soluble Cluster of Differentiation 14; **VEGFA**, Vascular Endothelial Growth Factor A; **FLT1**, fms related receptor tyrosine kinase 1; **VEGF**, Vascular Endothelial Growth Factor; **FCGR2B**, Fc Gamma Receptor II-b; **CRP**, C Reactive Protein; **CASP1**, Caspase 1; **IL-1 β**, Interleukin 1 Beta; **IL1R2**, Interleukin 1 Receptor type II; **ROBO4**, Roundabout Guidance Receptor 4.

## Data Availability

No new data were created or analyzed in this study. Data sharing is not applicable to this article.

## Supplementary Materials

The following supporting information can be downloaded at: https://www.mdpi.com/article/10.3390/ijms27052277/s1.

## References

[1] Snellings D.A., Hong C.C., Ren A.A., Lopez-Ramirez M.A., Girard R., Srinath A., Marchuk D.A., Ginsberg M.H., Awad I.A., Kahn L.K. (2021). Cerebral Cavernous Malformation: From Mechanism to Therapy. Circ. Res..

[2] Chohan M.O., Marchiò S., Morrison L.A., Sidman R.L., Cavenee W.K., Dejana E., Yonas H., Pasqualini R., Arap W. (2018). Emerging Pharmacologic Targets in Cerebral Cavernous Malformation and Potential Strategies to Alter the Natural History of a Difficult Disease A Review. JAMA Neurol..

[3] Retta S.F., Glading A.J. (2016). Oxidative stress and inflammation in cerebral cavernous malformation disease pathogenesis: Two sides of the same coin. Int. J. Biochem. Cell Biol..

[4] Marchi S., Trapani E., Corricelli M., Goitre L., Pinton P., Retta S.F. (2016). Beyond multiple mechanisms and a unique drug: Defective autophagy as pivotal player in cerebral cavernous malformation pathogenesis and implications for targeted therapies. Rare Dis..

[5] Cavalcanti D.D., Kalani M.Y.S., Martirosyan N.L., Eales J., Spetzler R.F., Preul M.C. (2012). Cerebral cavernous malformations: From genes to proteins to disease. J. Neurosurg..

[6] Riant F., Bergametti F., Ayrignac X., Boulday G., Tournier-Lasserve E. (2010). Recent insights into cerebral cavernous malformations: The molecular genetics CCM. FEBS J..

[7] Dubovsky J., Zabramski J.M., Kurth J., Spetzler R.F., Rich S.S., Orr H.T., Weber J.L. (1995). A gene responsible for cavernous malformations of the brain maps to chromosome 7q. Hum. Mol. Genet..

[8] Urat M., Ünel G., Wad S.A.A., Arin K., Inberg F., Ohn J., Batjer H.H., Kopitnik T.A., Morrison L., Giannotta S.L. (1996). A Founder Mutation as a Cause of Cerebral Cavernous Malformation in Hispanic Americans. N. Engl. J. Med..

[9] Akers A.L., Johnson E., Steinberg G.K., Zabramski J.M., Marchuk D.A. (2008). Biallelic somatic and germline mutations in cerebral cavernous malformations (CCMs): Evidence for a two-hit mechanism of CCM pathogenesis. Hum. Mol. Genet..

[10] Fauth C., Rostasy K., Rath M., Gizewski E., Lederer A.G., Sure U., Zschocke J., Felbor U. (2015). Highly variable intrafamilial manifestations of a CCM3 mutation ranging from acute childhood cerebral haemorrhage to late-onset meningiomas. Clin. Neurol. Neurosurg..

[11] Cutsforth-Gregory J.K., Lanzino G., Link M.J., Brown R.D., Flemming K.D. (2015). Characterization of radiation-induced cavernous malformations and comparison with a nonradiation cavernous malformation cohort. J. Neurosurg..

[12] Strenger V., Sovinz P., Lackner H., Dornbusch H.J., Lingitz H., Eder H.G., Moser A., Urban C. (2008). Intracerebral cavernous hemangioma after cranial irradiation in childhood: Incidence and risk factors. Strahlenther. Onkol..

[13] Gault J., Shenkar R., Recksiek P., Awad I.A. (2005). Biallelic Somatic and Germ Line CCM1 Truncating Mutations in a Cerebral Cavernous Malformation Lesion. Stroke.

[14] Gault J., Awad I.A., Recksiek P., Shenkar R., Breeze R., Handler M., Kleinschmidt-DeMasters B. (2009). Cerebral Cavernous Malformations. Neurosurgery.

[15] Pagenstecher A., Stahl S., Sure U., Felbor U. (2009). A two-hit mechanism causes cerebral cavernous malformations: Complete inactivation of CCM1, CCM2 or CCM3 in affected endothelial cells. Hum. Mol. Genet..

[16] Zhu Y., Wu Q., Fass M., Xu J.F., You C., Müller O., Sandalcioglu I.E., Jian-Min Z., Ulrich S. (2011). In vitro characterization of the angiogenic phenotype and genotype of the endothelia derived from sporadic cerebral cavernous malformations. Neurosurgery.

[17] Pilz R.A., Skowronek D., Mellinger L., Bekeschus S., Felbor U., Rath M. (2023). Endothelial Differentiation of CCM1 Knockout iPSCs Triggers the Establishment of a Specific Gene Expression Signature. Int. J. Mol. Sci..

[18] Abdelilah-Seyfried S., Tournier-Lasserve E., Derry W.B. (2020). Blocking Signalopathic Events to Treat Cerebral Cavernous Malformations. Trends Mol. Med..

[19] Ren A.A., Snellings D.A., Su Y.S., Hong C.C., Castro M., Tang A.T., Detter M.R., Hobson N., Girard R., Romanos S. (2021). PIK3CA and CCM mutations fuel cavernomas through a cancer-like mechanism. Nature.

[20] Plummer N.W., Gallione C.J., Srinivasan S., Zawistowski J.S., Louis D.N., Marchuk D.A. (2004). Loss of p53 Sensitizes Mice with a Mutation in Ccm1 (KRIT1) to Development of Cerebral Vascular Malformations. Am. J. Pathology. Discov. Basic Transl. Pathobiol..

[21] Boulday G., Rudini N., Maddaluno L., Blécon A., Arnould M., Gaudric A., Chapon F., Adams R.H., Dejana E., Tournier-Lasserve E. (2011). Developmental timing of CCM2 loss influences cerebral cavernous malformations in mice. J. Exp. Med..

[22] Whitehead K.J., Chan A.C., Navankasattusas S., Koh W., London N.R., Ling J., Mayo A.H., Drakos S.G., Jones C.A., Zhu W. (2009). The Cerebral Cavernous Malformation signaling pathway promotes vascular integrity via Rho GTPases. Nat. Med..

[23] Shenkar R., Peiper A., Pardo H., Moore T., Lightle R., Girard R., Hobson N., Polster S.P., Koskimäki J., Zhang D. (2019). Rho kinase inhibition blunts lesion development and hemorrhage in murine models of aggressive Pdcd10/Ccm3 disease. Stroke.

[24] Hong T., Xiao X., Ren J., Cui B., Zong Y., Zou J., Kou Z., Jiang N., Meng G., Zeng G. (2021). Somatic *MAP3K3* and *PIK3CA* mutations in sporadic cerebral and spinal cord cavernous malformations. Brain.

[25] Zhang J., Croft J., Le A. (2023). Familial CCM Genes Might Not Be Main Drivers for Pathogenesis of Sporadic CCMs-Genetic Similarity between Cancers and Vascular Malformations. J. Pers. Med..

[26] Abdulrauf S.I., Kaynar M.Y., Awad I.A. (1999). A comparison of the clinical profile of cavernous malformations with and without associated venous malformations. Neurosurgery.

[27] Brinjikji W., El-Rida El-Masri A., Wald J.T., Lanzino G. (2017). Prevalence of Developmental Venous Anomalies Increases with Age. Stroke.

[28] Snellings D.A., Girard R., Lightle R., Srinath A., Romanos S., Li Y., Chen C., Ren A.A., Kahn M.L., Awad I.M. (2022). Developmental venous anomalies are a genetic primer for cerebral cavernous malformations. Nat. Cardiovasc. Res..

[29] Huo R., Yang Y., Sun Y., Zhou Q., Zhao S., Mo Z., Xu H., Wang J., Weng J., Jiao W. (2023). Endothelial hyperactivation of mutant MAP3K3 induces cerebral cavernous malformation enhanced by PIK3CA GOF mutation. Angiogenesis.

[30] Weng J., Yang Y., Song D., Huo R., Li H., Chen Y., Nam Y., Zhou Q., Jiao Y., Fu W. (2021). Somatic MAP3K3 mutation defines a subclass of cerebral cavernous malformation. Am. J. Hum. Genet..

[31] You C., Erol Sandalcioglu I., Dammann P., Felbor U., Sure U., Zhu Y. (2013). Loss of CCM3 impairs DLL4-Notch signalling: Implication in endothelial angiogenesis and in inherited cerebral cavernous malformations. J. Cell Mol. Med..

[32] You C., Zhao K., Dammann P., Keyvani K., Kreitschmann-Andermahr I., Sure U., Zhu Y. (2017). EphB4 forward signalling mediates angiogenesis caused by CCM3/PDCD10-ablation. J. Cell Mol. Med..

[33] Saban D., Larisch J., Nickel A.C., Pierscianek D., Dammann P., Sure U., Zhu Y. (2020). DNA promoter methylation of CCM genes in human cerebral cavernous malformations: Importance of confirming MSP data through sequencing. Eur. J. Med. Genet..

[34] Ren J., Huang Y., Ren Y., Tu T., Qiu B., Ai D., Bai Z., Li F., Chen X., Feng Z. (2023). Somatic variants of *MAP3K3* are sufficient to cause cerebral and spinal cord cavernous malformations. Brain.

[35] Zhou Z., Tang A.T., Wong W.Y., Bamezai S., Goddard L.M., Shenkar R., Zhou S., Yang J., Wright A.C., Foley W. (2016). Cerebral cavernous malformations arise from endothelial gain of MEKK3-KLF2/4 signaling HHS Public Access. Nature.

[36] Zhou Z., Rawnsley D.R., Zheng X., Kahn M.L. (2015). The Cerebral Cavernous Malformation Pathway Controls Cardiac Development via Regulation of Endocardial MEKK3 Signaling and KLF Expression. Dev. Cell.

[37] Ayata C., Kim H., Morrison L., Liao J.K., Gutierrez J., Lopez-Toledano M., Carrazana E., Rabinowicz A., Issam A.A. (2024). Role of Rho-Associated Kinase in the Pathophysiology of Cerebral Cavernous Malformations. Neurol. Genet..

[38] Vannier D.R., Shapeti A., Chuffart F., Planus E., Manet S., Rivier P., Destaing O., Albiges-Rizo C., Oosterwyck H., Faurobert E. (2021). CCM2-deficient endothelial cells undergo a ROCK-dependent reprogramming into senescence-associated secretory phenotype. Angiogenesis.

[39] Santamaria S., De Groot R. (2020). ADAMTS proteases in cardiovascular physiology and disease. Open Biol..

[40] Hong C.C., Tang A.T., Detter M.R., Choi J.P., Wang R., Yang X., Guerrero A.A., Hobson N., Girard R., Lightle R. (2020). Cerebral cavernous malformations are driven by ADAMTS5 proteolysis of versican. J. Exp. Med..

[41] Zebda N., Dubrovskyi O., Birukov K.G. (2011). Focal adhesion kinase regulation of mechanotransduction and its impact on endothelial cell functions. Microvasc. Res..

[42] Lamalice L., Le Boeuf F., Huot J. (2007). Endothelial Cell Migration During Angiogenesis. Circ. Res..

[43] Santos A.N., Rauschenbach L., Saban D., Chen B., Darkwah Oppong M., Herten A., Gull H.H., Rieß C., Deuschl C., Schmidt B. (2022). Multiple cerebral cavernous malformations: Clinical course of confirmed, assumed and non-familial disease. Eur. J. Neurol..

[44] Shenkar R., Shi C., Rebeiz T., Stockton R.A., McDonald D.A., Mikati A.G., Zhang L., Austin C., Akers A.L., Gallione C.J. (2015). Exceptional aggressiveness of cerebral cavernous malformation disease associated with PDCD10 mutations. Genet. Med..

[45] Tang A.T., Sullivan K.R., Hong C.C., Goddard L.M., Mahadevan A., Ren A., Pardo H., Peiper A., Griffin E., Tanes C. (2019). Distinct cellular roles for PDCD10 define a gut-brain axis in cerebral cavernous malformation. Sci. Transl. Med..

[46] Denier C., Labauge P., Bergametti F., Marchelli F., Riant F., Arnoult M., Maciazek J., Vicaut E., Brunereau L., Tournier-Lasserve M. (2006). Genotype-phenotype correlations in cerebral cavernous malformations patients. Ann. Neurol..

[47] Fung T.C., Olson C.A., Hsiao E.Y. (2017). Interactions between the microbiota, immune and nervous systems in health and disease HHS Public Access. Nat. Neurosci..

[48] Sharon G., Garg N., Debelius J., Knight R., Dorrestein P.C., Mazmanian S.K. (2014). Cell Metabolism Specialized Metabolites from the Microbiome in Health and Disease. Cell Metab..

[49] Tang A.T., Choi J.P., Kotzin J.J., Yang Y., Hong C.C., Hobson N., Girard R., Zeineddine H.A., Lightle R., Moore T. (2017). Endothelial TLR4 and the microbiome drive cerebral cavernous malformations. Nature.

[50] González-Gallardo E., Rauschenbach L., Santos A.N., Riess C., Li Y., Tippelt S., Marina A.D., Dohna-Schwake C., Sure U., Dammann P. (2023). Giant Cavernous Malformation Mimicking an Infiltrative Intracranial Neoplasm in Children–Case Report and Systematic Review of the Literature. World Neurosurg..

[51] Ozgen B., Senocak E., Oguz K.K., Soylemezoglu F., Akalan N. (2010). Radiological features of childhood giant cavernous malformations. Neuroradiology.

[52] Clatterbuck R.E., Moriarity J.L., Elmaci I., Lee R.R., Breiter S.N., Rigamonti D. (2000). Dynamic nature of cavernous malformations: A prospective magnetic resonance imaging study with volumetric analysis. J. Neurosurg..

[53] Lopez-Ramirez M.A., Pham A., Girard R., Wyseure T., Hale P., Yamashita A., Koskimäki J., Polster S., Saadat L., Romero I.A. (2019). Cerebral cavernous malformations form an anticoagulant vascular domain in humans and mice. Blood.

[54] DiStefano P.V., Glading A.J. (2020). VEGF signalling enhances lesion burden in KRIT1 deficient mice. J. Cell Mol. Med..

[55] Globisch M.A., Onyeogaziri F.C., Jauhiainen S., Yau A.C.Y., Orsenigo F., Conze L.L., Arce M., Corada M., Smith R.O., Rorsman C. Immunothrombosis and Vascular Heterogeneity in Cerebral Cavernous Malformation. http://ashpublications.org/blood/article-pdf/140/20/2154/2054622/blood_bld-2021-015350-main.pdf.

[56] Neubauer K., Zieger B. (2022). Endothelial cells and coagulation. Cell Tissue Res..

[57] Globisch M.A., Onyeogaziri C., Smith R.O., Arce M., Magnusson P.U. (2022). Dysregulated Hemostasis and Immunothrombosis in Cerebral Cavernous Malformations. Int. J. Mol. Sci..

[58] Much C.D., Sendtner B.S., Schwefel K., Freund E., Bekeschus S., Otto O., Pagenstecher A., Felbor U., Rath M., Spiegler S. (2021). Inactivation of Cerebral Cavernous Malformation Genes Results in Accumulation of von Willebrand Factor and Redistribution of Weibel-Palade Bodies in Endothelial Cells. Front. Mol. Biosci..

[59] Sforza D.M., Putman C.M., Cebral J.R. (2009). Hemodynamics of Cerebral Aneurysms. Annu. Rev. Fluid Mech..

[60] Tang H., Wang Q., Xu F., Zhang X., Zeng Z., Yan Y., Lu Z., Xue G., Zuo Q., Luo Y. (2021). Underlying mechanism of hemodynamics and intracranial aneurysm. Chin. Neurosurg. J..

[61] Li Y., Srinath A., Alcazar-Felix R., Hage S., Bindal A., Lightle R., Shenkar R., Shi C., Girard R., Issam A.A. (2023). Inflammatory Mechanisms in a Neurovascular Disease: Cerebral Cavernous Malformation. Brain Sci..

[62] Girard R., Zeineddine H.A., Koskimäki J., Fam M.D., Cao Y., Shi C., Moore T., Lightle R., Stadnik A., Chaudagar K. (2018). Plasma Biomarkers of Inflammation and Angiogenesis Predict Cerebral Cavernous Malformation Symptomatic Hemorrhage or Lesional Growth HHS Public Access. Circ. Res..

[63] Girard R., Li Y., Stadnik A., Shenkar R., Hobson N., Romanos S., Sharbel B.A., Srinath A., Thomas B.S., Lightle R. (2021). A Roadmap for Developing Plasma Diagnostic and Prognostic Biomarkers of Cerebral Cavernous Angioma with Symptomatic Hemorrhage (CASH). Neurosurgery.

[64] Srinath A., Xie B., Li Y., Sone J.Y., Romanos S., Chen C., Sharma A., Polster S., Dorrestein P.C., Weldon K.C. (2023). Plasma metabolites with mechanistic and clinical links to the neurovascular disease cavernous angioma. Commun. Med..

[65] Horne M.A., Flemming K.D., Su I.C., Stapf C., Jeon J.P., Li D., Maxwell S.S., White P., Christianson T.J., Agid R. (2016). Clinical course of untreated cerebral cavernous malformations: A meta-analysis of individual patient data. Artic. Lancet Neurol..

[66] Santos A.N., Rauschenbach L., Gull H.H., Olbrich A., Lahl K., Darkwah Oppong M., Dinger T.F., Rieß C., Chen B., Lenkeit A. (2023). Central nervous system cavernous malformations: Cross-sectional study assessing rebleeding risk after a second haemorrhage. Eur. J. Neurol..

[67] Lazzaroni F., Meessen J.M.T.A., Sun Y., Lanfranconi S., Scola E., D’Alessandris Q.G., Tassig L., Carrieroh M.R., Castorii M., Marino S. (2024). Circulating biomarkers in familial cerebral cavernous malformation. EBioMedicine.

[68] Page M.J., McKenzie J.E., Bossuyt P.M., Boutron I., Hoffmann T.C., Mulrow C.D., Shamseer L., Tetzlaff J.M., A Akl E., Brennan S.E. (2021). The PRISMA 2020 statement: An updated guideline for reporting systematic reviews. BMJ.

[69] Girard R., Khanna O., Shenkar R., Zhang L., Wu M., Jesselson M., Zeineddine H.A., Gangal A., Fam M.D., Gibson C.C. (2016). Peripheral plasma Vitamin D and non-HDL cholesterol reflect the severity of cerebral cavernous malformation disease. Biomark. Med..

[70] Gibson C.C., Zhu W., Davis C.T., Bowman-Kirigin J.A., Chan A.C., Ling J., Walker A.E., Goitre L., Monache S.D., Retta S.F. (2015). Strategy for Identifying Repurposed Drugs for the Treatment of Cerebral Cavernous Malformation HHS Public Access. Circulation.

[71] Venugopal V., Sumi S. (2022). Molecular biomarkers and drug targets in brain arteriovenous and cavernous malformations: Where are we?. Stroke.

[72] Flemming K.D., Kumar S., Brown R.D., Singh R.J., Whitehead K., McCreath L., Lanzino G. (2020). Cavernous Malformation Hemorrhagic Presentation at Diagnosis Associated with Low 25-Hydroxy-Vitamin D Level. Cerebrovasc. Dis..

[73] Keep R.F., Zhou N., Xiang J., Andjelkovic A.V., Hua Y., Xi G. (2014). Vascular disruption and blood-brain barrier dysfunction in intracerebral hemorrhage. Fluids Barriers CNS.

[74] Luissint A.C., Artus C., Glacial F., Ganeshamoorthy K., Couraud P.O. (2012). Tight junctions at the blood brain barrier: Physiological architecture and disease-associated dysregulation. Fluids Barriers CNS.

[75] Gao Y., Zhao Z., Yang L., Liu X., Xing X., Zhang H., Yun J., Ou X., Su X., Lu X. (2017). Arsenic exposure assists ccm3 genetic polymorphism in elevating blood pressure. Oncotarget.

[76] Hsuchou H., Kastin A.J., Mishra P.K., Pan W. (2012). C-reactive protein increases BBB permeability: Implications for obesity and neuroinflammation. Cell Physiol. Biochem..

[77] Chen Y., Hao Q., Kim H., Su H., Letarte M., Karumanchi S.A., Lawton M.T., Barbaro N.M., Yang G.-Y., Young W.L. (2009). Soluble endoglin modulates aberrant cerebral vascular remodeling. Ann. Neurol..

[78] Mcallister K.A., Grogg K.M., Johnson D.W., Gallionel C.J., Baldwin M.A., Jackson C.E., Helmbold E.A., Markel D.S., McKinnon W.C., Murrel J. (1994). Endoglin, a TGF-P binding protein of endothelial cells, is the gene for hereditary haemorrhagic telangiectasia type 1. Nat. Genet..

[79] Lopez-Ramirez M.A., Fonseca G., Zeineddine H.A., Girard R., Moore T., Pham A., Cao Y., Shenkar R., Kreuk B., Lagarrigue F. (2017). Thrombospondin1 (TSP1) replacement prevents cerebral cavernous malformations. J. Exp. Med..

[80] Chehuen Bicalho V., da Fontoura Galvão G., Lima Fontes-Dantas F., Paulo da Costa Gonçalves J., Dutra de Araujo A., Carolina França L., Leite P.E.C., Campolina Vidal C., Castro Filho R., Alves-Leon S.V. (2021). Asymptomatic cerebral cavernous angiomas associated with plasma marker signature. J. Clin. Neurosci..

[81] Kar S., Perrelli A., Bali K.K., Mastrocola R., Kar A., Khan B., Gand L., Nayak A., Hartmann C., Kunz W.S. (2024). Identification of galectin-3 as a novel potential prognostic/predictive biomarker and therapeutic target for cerebral cavernous malformation disease. Genes Dis..

[82] Girard R., Zeineddine H.A., Fam M.D., Mayampurath A., Cao Y., Shi C., Shenkar R., Polster S.P., Jesselson M., Duggan R. (2018). Plasma Biomarkers of Inflammation Reflect Seizures and Hemorrhagic Activity of Cerebral Cavernous Malformations HHS Public Access. Transl. Stroke Res..

[83] Ng C.T., Fong L.Y., Sulaiman M.R., Moklas M.A.M., Yong Y.K., Hakim M.N., Ahmad Z. (2015). Interferon-Gamma Increases Endothelial Permeability by Causing Activation of p38 MAP Kinase and Actin Cytoskeleton Alteration. J. Interferon Cytokine Res..

[84] Pawlikowska L., Tran M.N., Achrol A.S., McCulloch C.E., Ha C., Lind D.L., Hashimoto T., Zaroff J., Lawton M.T., Marchuk D.A. (2004). Polymorphisms in genes involved in inflammatory and angiogenic pathways and the risk of hemorrhagic presentation of brain arteriovenous malformations. Stroke.

[85] Jauhiainen S., Onyeogaziri F., Savander H., Lazzaroni F., Liu Conze L., Laakso A., Niemelä M., Dejana E., Rezai B., Magnusson P. (2022). O-048 Proteomics analysis on human cerebral cavernous malformations reveals novel biomarkers for the disease pathology. J. NeuroInterv. Surg..

[86] Lampugnani M.G., Dejana E., Giampietro C. (2018). Vascular endothelial (VE)-cadherin, endothelial adherens junctions, and vascular disease. Cold Spring Harb. Perspect. Biol..

[87] Robinson J.R., Awad I.A., Zhou P., Barna B.P., Estes M.L. (1995). Expression of basement membrane and endothelial cell adhesion molecules in vascular malformations of the brain: Preliminary observations and working hypothesis. Neurol. Res..

[88] Jauhiainen S., Onyeogaziri F.C., Lazzaroni F., Conze L.L., Laakkonen J.P., Laham-Karam N., Laakso A., Niemelä M., Jahromi B.R., Magnusson P.U. (2024). Proteomics on human cerebral cavernous malformations reveals novel biomarkers in neurovascular dysfunction for the disease pathology. Biochim. Biophys. Acta Mol. Basis Dis..

[89] Shenkar R., Shi C., Austin C., Moore T., Lightle R., Cao Y., Zhang L., Wu M., Zeineddine H.A., Girard R. (2017). RhoA Kinase Inhibition with Fasudil Versus Simvastatin in Murine Models of Cerebral Cavernous Malformations. Stroke.

[90] Bicer A., Guclu B., Ozkan A., Kurtkaya O., Koc Y., Necmettin Pamir M., Kilic T. (2010). Laboratory Study Expression of angiogenesis associated matrix metalloproteinases and extracellular matrix proteins in cerebral vascular malformations. Clin. Neurosci..

[91] Polster S.P., Sharma A., Tanes C., Tang A.T., Mericko P., Cao Y., Carrión-Penagos J., Girard R., Koskimäki J., Zhang D. (2020). Permissive microbiome characterizes human subjects with a neurovascular disease cavernous angioma. Nat. Commun..

[92] Bergstrom K.S.B., Kissoon-Singh V., Gibson D.L., Ma C., Montero M., Sham H.P., Ryz N., Huang T., Velcich A., Finlay B.B. (2010). Muc2 Protects against Lethal Infectious Colitis by Disassociating Pathogenic and Commensal Bacteria from the Colonic Mucosa. PLoS Pathog..

[93] Chassaing B., Srinivasan G., Delgado M.A., Young A.N., Gewirtz A.T., Vijay-Kumar M. (2012). Fecal lipocalin 2, a sensitive and broadly dynamic non-invasive biomarker for intestinal inflammation. PLoS ONE.

[94] Wüstehube J., Bartol A., Liebler S.S., Brütsch R., Zhu Y., Felbor U., Sure U., Augustin H.G., Fischer A. (2010). Cerebral cavernous malformation protein CCM1 inhibits sprouting angiogenesis by activating DELTA-NOTCH signaling. Proc. Natl. Acad. Sci. USA.

[95] Jones C.A., London N.R., Chen H., Park K.W., Sauvaget D., Stockton R.A., Wythe J.D., Larrieu-Lahargue F., Mukouyama Y.-S., Lindblom P. (2008). Robo4 stabilizes the vascular network by inhibiting pathologic angiogenesis and endothelial hyperpermeability. Nat. Med..

[96] Choquet H., Pawlikowska L., Nelson J., Mcculloch C.E., Akers A., Baca B., Khan Y., Hart B., Morrison L., Kim H. (2014). Polymorphisms in inflammatory and immune response genes associated with cerebral cavernous malformation type 1 severity on behalf of the Brain Vascular Malformation Consortium (BVMC) Study. Cerebrovasc. Dis..

[97] Wetzel-Strong S.E., Weinsheimer S., Nelson J., Pawlikowska L., Clark D., Starr M.D., Liu Y., Kim H., Faughnan M.E., Nixon A.B. (2021). Pilot investigation of circulating angiogenic and inflammatory biomarkers associated with vascular malformations. Orphanet J. Rare Dis..

[98] Cunha S.I., Magnusson P.U., Dejana E., Lampugnani M.G. (2017). Deregulated TGF-β/BMP signaling in vascular malformations. Circ. Res..

[99] Jung K.H., Chu K., Jeong S.W., Park H.K., Bae H.J., Yoon B.W. (2003). Cerebral Cavernous Malformations with Dynamic and Progressive Course Correlation Study with Vascular Endothelial Growth Factor. Arch. Neurol..

[100] Lyne S.B., Girard R., Koskimäki J., Zeineddine H.A., Zhang D., Cao Y., Li Y., Stadnik A., Moore T., Lightle R. (2019). Biomarkers of cavernous angioma with symptomatic hemorrhage. JCI Insight.

[101] Florian I.A., Buruiana A., Timis T.L., Susman S., Florian I.S., Balasa A., Berindan-Neagoe I. (2021). An Insight into the microRNAs Associated with Arteriovenous and Cavernous Malformations of the Brain. Cells.

[102] Croft J., Grajeda B., Aguirre L.A., Abou-Fadel J.S., Ellis C.C., Estevao I., Almeida I.C., Zhang J. (2024). Circulating Blood Prognostic Biomarker Signatures for Hemorrhagic Cerebral Cavernous Malformations (CCMs). Int. J. Mol. Sci..

[103] Galvao G., Trefilio L., Salvio A., Silva E., Alves-Leon S., Fontes-Dantas F.L., Almeida I.C., Zhang J. (2024). Genetic Markers and Predictive Factors Influencing the Aggressive Behavior of Cerebral Cavernous Malformation. medRxiv.

[104] Kar S., Bali K.K., Baisantry A., Geffers R., Hartmann C., Samii A., Bertalanffy H. (2018). Genome-Wide Sequencing Reveals Small Nucleolar RNAs Downregulated in Cerebral Cavernous Malformations. Cell Mol. Neurobiol..

[105] Kim H., Flemming K.D., Nelson J.A., Lui A., Majersik J.J., Dela Cruz M., Zabramski J., Trevizo O., Lanzino G., Zafar A. (2021). Baseline Characteristics of Patients with Cavernous Angiomas with Symptomatic Hemorrhage in Multisite Trial Readiness Project. Stroke.

[106] Sone J.Y., Li Y., Hobson N., Romanos S.G., Srinath A., Lyne S.B., Shkoukani A., Carrion-Penagos J., Stadnik A., Piedad K. (2021). Perfusion and permeability as diagnostic biomarkers of cavernous angioma with symptomatic hemorrhage. J. Cereb. Blood Flow Metab..

[107] Girard R., Fam M.D., Zeineddine H.A., Tan H., Mikati A.G., Shi C., Jesselson M., Shenkar R., Wu M., Cao Y. (2017). Vascular permeability and iron deposition biomarkers in longitudinal follow-up of cerebral cavernous malformations. J. Neurosurg..

[108] Mikati A.G., Khanna O., Zhang L., Girard R., Shenkar R., Guo X., Shah A., Larsson H.B.W., Tan H., Li L. (2015). Vascular permeability in cerebral cavernous malformations. J. Cereb. Blood Flow Metab..

[109] Hage S., Kinkade S., Girard R., Flemming K.D., Kim H., Torbey M.T., Huang J., Huston J., Shu Y., Selwyn R.G. (2023). Cavernous Angioma Symptomatic Hemorrhage (CASH) Trial Readiness II: Imaging Biomarkers and Trial Modeling. medRxiv.

[110] Zeineddine H.A., Girard R., Cao Y., Hobson N., Fam M.D., Stadnik A., Tan H., Shen J., Chaudagar K., Shenkar R. (2018). Quantitative susceptibility mapping as a monitoring biomarker in cerebral cavernous malformations with recent hemorrhage. J. Magn. Reson. Imaging.

[111] Tan H., Zhang L., Mikati A.G., Girard R., Khanna O., Fam M.D., Liu Y., Wang Y., Edelman R.R., Christoforidis G. (2016). Quantitative susceptibility mapping in cerebral cavernous malformations: Clinical correlations. Am. J. Neuroradiol..

[112] Mikati A.G., Tan H., Shenkar R., Li L., Zhang L., Guo X., Larsson H.B.W., Shu C., Liu T., Wang Y. (2014). Dynamic Permeability and Quantitative Susceptibility. Stroke.

[113] Rauschenbach L., Santos A.N., Gull H.H., Rieß C., Deuschl C., Schmidt B., Darkwah Oppong M., Gembruch O., Özkan N., Jabbarli R. (2022). Functional impact of multiple bleeding events in patients with conservatively treated spinal cavernous malformations. J. Neurosurg. Spine.

[114] Garcia J.M., Stillings S.A., Leclerc J.L., Phillips H., Edwards N.J., Robicsek S.A., Hoh B.L., Blackburn S., Dore S. (2017). Role of interleukin-10 in acute brain injuries. Front. Neurol..

[115] Ip W.K.E., Hoshi N., Shouval D.S., Snapper S., Medzhitov R. (2017). Anti-inflammatory effect of IL-10 mediated by metabolic reprogramming of macrophages. Science.

[116] Mosser D.M., Edwards J.P. (2008). Exploring the full spectrum of macrophage activation. Nat. Rev. Immunol..

[117] Mineo C., Gormley A.K., Yuhanna I.S., Osborne-Lawrence S., Gibson L.L., Hahner L., Shohet R.V., Black S., Salmon J.E., Samols D. (2005). FcγRIIB mediates C-reactive protein inhibition of endothelial NO synthase. Circ. Res..

[118] Stein M.P., Mold C., Du Clos T.W. (2000). C-Reactive Protein Binding to Murine Leukocytes Requires Fcγ Receptors. J. Immunol..

[119] Lévêque M., Jeune K.S.L., Jouneau S., Moulis S., Desrues B., Belleguic C., Brinchault G., Le Trionnaire S., Gangneux J.-P., Dimanche-Boitrel M.-T. (2017). Soluble CD14 acts as a DAMP in human macrophages: Origin and involvement in inflammatory cytokine/chemokine production. FASEB J..

[120] Lloyd-Jones K.L., Kelly M.M., Kubes P. (2008). Varying Importance of Soluble and Membrane CD14 in Endothelial Detection of Lipopolysaccharide. J. Immunol..

[121] Abe T., Morishige M., Ooba H., Kamida T., Fujiki M., Kobayashi H., Sakoda T., Kimba Y. (2009). The association between high VEGF levels and multiple probable punctuate cavernous malformations. Acta Neurochir..

[122] Yadav S.S., Narayan G. (2014). Role of ROBO4 signalling in developmental and pathological angiogenesis. BioMed Res. Int..

[123] Sokol H., Pigneur B., Watterlot L., Lakhdari O., Bermúdez-Humarán L.G., Gratadoux J.J., Blugeon S., Bridonneau C., Furet J.-P., Corthier G. (2008). From the Cover: *Faecalibacterium prausnitzii* is an anti-inflammatory commensal bacterium identified by gut microbiota analysis of Crohn disease patients. Proc. Natl. Acad. Sci. USA.

[124] Miquel S., Martín R., Rossi O., Bermú Dez-Humará L.G., Chatel J.M., Sokol H., Thomas M., Wells J.M., Langella P. (2013). *Faecalibacterium prausnitzii* and human intestinal health. Curr. Opin. Microbiol..

[125] O’Callaghan A., van Sinderen D. (2016). Bifidobacteria and Their Role as Members of the Human Gut Microbiota. Front. Microbiol..

[126] Haran J.P., Bhattarai S.K., Foley S.E., Dutta P., Ward D.V., Bucci V., McCormick B. (2024). Alzheimer’s Disease Microbiome Is Associated with Dysregulation of the Anti-Inflammatory P-Glycoprotein Pathway. mBio.

[127] Xi J., Ding D., Zhu H., Wang R., Su F., Wu W., Xiao Z., Liang X., Zhao Q., Hong Z. (2021). Disturbed microbial ecology in Alzheimer’s disease: Evidence from the gut microbiota and fecal metabolome. BMC Microbiol..

[128] Langkammer C., Liu T., Khalil M., Enzinger C., Jehna M., Fuchs S., Fazekas F., Wang Y., Ropele S. (2013). Quantitative Susceptibility Mapping in Multiple Sclerosis. Radiology.

[129] Liu T., Surapaneni K., Lou M., Cheng L., Spincemaille P., Wang Y. (2012). Cerebral Microbleeds: Burden Assessment by Using Quantitative Susceptibility Mapping 1. Radiology.

[130] Fritzsch D., Reiss-Zimmermann M., Trampel R., Turner R., Hoffmann K.T., Schäfer A. (2014). Seven-tesla magnetic resonance imaging in wilson disease using quantitative susceptibility mapping for measurement of copper accumulation. Investig. Radiol..

[131] Akers A., Al-Shahi Salman R., Edin F., Awad I.A., Dahlem K., Flemming K., Blaine H., Helen K., Ignacio J.-T., Douglas K. (2017). CCM CARE GUIDELINES Synopsis of Guidelines for the Clinical Management of Cerebral Cavernous Malformations: Consensus Recommendations Based on Systematic Literature Review by the Angioma Alliance Scientific Advisory Board Clinical Experts Panel. Neurosurgery.

[132] Zhang Z.G., Zhang L., Jiang Q., Zhang R., Davies K., Powers C., Bruggen N., Chopp M. (2000). VEGF enhances angiogenesis and promotes blood-brain barrier leakage in the ischemic brain. J. Clin. Investig..

[133] Prabhakaran S., Naidech A.M. (2012). Progress Review Ischemic Brain Injury After Intracerebral Hemorrhage A Critical Review. Stroke.

