# Supplementary material for: Molecular Pathways and Circulating Biomarkers in Cerebral Cavernous Malformations—A Systematic Review
Source: Int J Mol Sci. 2026 Feb 28;27(5):2277. doi: 10.3390/ijms27052277 (PMC12985414; doi:10.3390/ijms27052277)
Supplement: Supplementary file 1 [file ijms-27-02277-s001.zip › ijms-4119759-supplementary/Figure S1- Cerebral Cavernous Malformation Genetical Identified Loci_07_12_2024.docx]

**Figure S1:** Cerebral Cavernous Malformation Genetical Identified Loci.


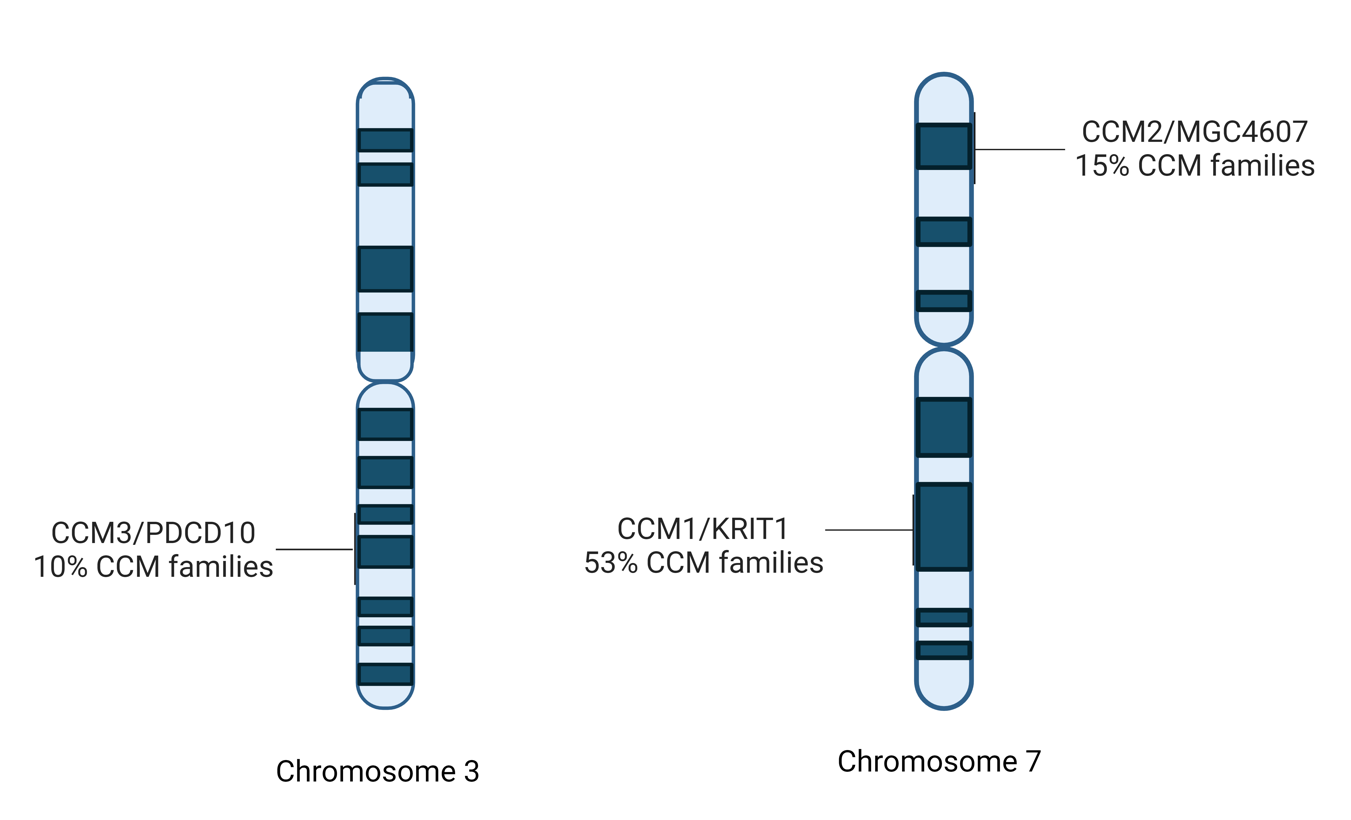


**Figure S1.** Cerebral Cavernous Malformation genetical identified loci.

**CCM1/KRIT1**, Krev Interaction Trapped Protein 1; **CCM2/MGC4607**, Malcaverin Protein; **CCM3/PDCD10**, Programmed Cell Death 10 Protein; Modified from Riant F, et al. 2010.
