# Supplementary material for: Molecular Pathways and Circulating Biomarkers in Cerebral Cavernous Malformations—A Systematic Review
Source: Int J Mol Sci. 2026 Feb 28;27(5):2277. doi: 10.3390/ijms27052277 (PMC12985414; doi:10.3390/ijms27052277)
Supplement: Supplementary file 1 [file ijms-27-02277-s001.zip › ijms-4119759-supplementary/Table S1- Conceptual Biomarker Definitions_07_12_2024.docx]

**Table S1:** Glossary of Conceptual Biomarker Definitions.

| Category | Definition |
| --- | --- |
| Diagnostic Biomarker | Protein/cytokine useful to detect the presence of a specific medical condition. |
| Prognostic Biomarker | Assesses the likelihood of progression, regression, or recurrence of this medical condition. |
| Monitoring Biomarker | Evaluates the progression or stability of medical conditions, as well as the effectiveness of an intervention over a defined period. |
| Predictive Biomarker | Identifies cases with an increased probability to develop an outcome upon exposure to certain medical intervention or to a specific environmental factor. |
| Susceptibility/Risk Biomarker | Reflect the probability of future development of a specific medical condition. |
| Safety Biomarker | Indicates or predicts toxicity or an adverse effect before or after a medical intervention. |

*Adapted from Girard et al. 2021.
