# Supplementary material for: Molecular Pathways and Circulating Biomarkers in Cerebral Cavernous Malformations—A Systematic Review
Source: Int J Mol Sci. 2026 Feb 28;27(5):2277. doi: 10.3390/ijms27052277 (PMC12985414; doi:10.3390/ijms27052277)
Supplement: Supplementary file 1 [file ijms-27-02277-s001.zip › ijms-4119759-supplementary/Table S2- Supplementary Table. Full Boolean Search Strategy..docx]

**Table S2:** Supplementary Table. Full Boolean Search Strategy.

| Database | Search Strategy Type | Search String | Date |
| --- | --- | --- | --- |
| PubMed (MEDLINE) | MeSH + Title/Abstract keywords | ("Cerebral Cavernous Malformations"[Mesh]  OR "cerebral cavernous malformation*"[tiab]  OR "cavernous malformation*"[tiab]  OR cavernoma*[tiab]  OR "cerebral cavernoma*"[tiab]  OR "cavernous angioma*"[tiab]  OR "cavernous hemangioma*"[tiab])  AND  ("Biomarkers"[Mesh]  OR biomarker*[tiab]  OR "biological marker*"[tiab]  OR "plasma biomarker*"[tiab]  OR "circulating biomarker*"[tiab]  OR "blood biomarker*"[tiab]  OR "serum biomarker*"[tiab])  AND  (plasma[tiab]  OR serum[tiab]  OR blood[tiab]  OR circulating[tiab]) | 15 Jan 2024 |
| Embase (Ovid) | Emtree + ti/ab/kw keywords | 1. exp cerebral cavernous malformation/  2. exp cavernous hemangioma/  3. (cerebral adj2 cavernous adj2 malformation*).ti,ab,kw.  4. (cavernous adj2 malformation*).ti,ab,kw.  5. cavernoma*.ti,ab,kw.  6. (cerebral adj2 cavernoma*).ti,ab,kw.  7. (cavernous adj2 angioma*).ti,ab,kw.  8. 1 or 2 or 3 or 4 or 5 or 6 or 7  9. exp biological marker/  10. biomarker*.ti,ab,kw.  11. (biological adj2 marker*).ti,ab,kw.  12. (plasma adj2 biomarker*).ti,ab,kw.  13. (circulating adj2 biomarker*).ti,ab,kw.  14. (blood adj2 biomarker*).ti,ab,kw.  15. (serum adj2 biomarker*).ti,ab,kw.  16. 9 or 10 or 11 or 12 or 13 or 14 or 15  17. exp blood/  18. exp plasma/  19. exp serum/  20. (blood or plasma or serum or circulating).ti,ab,kw.  21. 17 or 18 or 19 or 20  22. 8 and 16 and 21 | 30 Jan 2024 |
| Google Scholar | Keywords  (documented query) | ("cerebral cavernous malformation" OR "cavernous malformation" OR cavernoma OR "cerebral cavernoma" OR "cavernous angioma")  (biomarker OR "plasma biomarker" OR "circulating biomarker" OR "blood biomarker" OR "serum biomarker") | 24 Feb 2024 |
| Cochrane Library | MeSH descriptors + ti/ab/kw keywords | (MeSH descriptor: [Cerebral Cavernous Malformations] explode all trees  OR ("cerebral cavernous malformation*" OR "cavernous malformation*" OR cavernoma* OR "cerebral cavernoma*" OR "cavernous angioma*"):ti,ab,kw)  AND  (MeSH descriptor: [Biomarkers] explode all trees  OR (biomarker* OR "plasma biomarker*" OR "circulating biomarker*" OR "blood biomarker*" OR "serum biomarker*"):ti,ab,kw)  AND  (plasma OR serum OR blood OR circulating):ti,ab,kw | 31 Mar 2024 |
